# Supplementary material for: Cross-Modal Data Programming Enables Rapid Medical Machine Learning
Source: Patterns (N Y). 2020 Apr 28;1(2):100019. doi: 10.1016/j.patter.2020.100019 (PMC7413132; doi:10.1016/j.patter.2020.100019)
Supplement: Document S1. Supplemental Experimental Procedures and Tables S1–S4 [file mmc1.pdf]

PATTER, Volume *1*

Supplemental Information

Cross-Modal Data Programming

Enables Rapid Medical Machine Learning

Jared A. Dunnmon, Alexander J. Ratner, Khaled Saab, Nishith Khandwala, Matthew Markert, Hersh Sagreiya, Roger Goldman, Christopher Lee-Messer, Matthew P. Lungren, Daniel L. Rubin, and Christopher Ré

Supplemental Information

Supplemental Data Items

Dataset	CXR	EXR	HCT	EEG
Large FS vs. Large DP	0.3821	0.012	0.6223	0.0002
Medium FS vs. Large DP	0.0004	0.0002	0.0006	0.2670

Table S1. Statistical analysis of cross-modal data programming versus full supervision. P-values from the two-tailed DeLong non-parametric test[36] comparing ROC curves from median models. These results demonstrate that median CXR and HCT models supervised with cross-modal data programming (DP) are not statistically distinguishable from those supervised with the Large fully supervised (i.e. hand-labeled) set ($p > 0.35$), and are significantly different (DeLong $p < 0.001$) than those supervised with the Medium fully supervised set (see Table 1 for dataset size definitions). For EXR, median models supervised with cross-modal data programming were statistically superior to those trained using the Large fully supervised set (DeLong $p < 0.05$). For EEG, median models supervised with cross-modal data programming were statistically different than those supervised using the Large supervised set (DeLong $p < 0.05$), but not distinguishable from those trained using the Medium fully supervised set (DeLong $p > 0.25$).

Dataset	CXR	EXR	HCT	EEG
Metric	ROC-AUC	ROC-AUC	ROC-AUC	Weighted F1
Literature	0.95[7]	0.93[32]	0.96[6]	0.88[50]
Ours	0.95	0.91	0.92	0.85

Table S2. Comparison of fully supervised models to studies in the literature. Performance of fully supervised models presented in this work compared to best reported values from similar studies in the literature. For CXR, our results are equivalent to those of Dunnmon et al.,[7] for EXR our results are within 2 points ROC-AUC of Rajpurkar et al.,[32] for HCT our results are within 4 points ROC-AUC of Lee et al.,[6] and our results are within 3 points weighted F1 of Asif et al.[50] for EEG. Comparisons for CXR and EXR are direct, as similar datasets and labeling schema were used. Comparisons to HCT are conservative, as our model uses scan-level supervision, while Lee et al.[6] uses far more detailed slice-level supervision; the most relevant work using scan-level supervision for ICH detection is that of Jnawali et al.,[51] which attains an ROC-AUC of 0.86. In EEG, most studies report results using small numbers of patients;[37] modern studies using the Temple University Hospital EEG corpus are most relevant to our work,[33,50,52] but use costly retrospective multi-class supervision rather than binary technician labels, which are commonly obtained in the course of clinical practice.

Dataset	CXR	EXR	HCT	EEG
Comparable FS Size	Large	Large	Large	Medium
FS Labeling Time	9 Months	5 Months	3 Months	4 Months
DP Labeling Time	13 Hours	13 Hours	27 Hours	49 Hours
Percent of FS Time for DP	1.0%	1.8%	6.1%	7.9%

Table S3. Labeling times for comparable fully and weakly supervised models. We present labeling times for comparable models trained with full hand-labeled supervision (FS) and cross-modal data programming (DP). In each case, the DP model trained on the Large dataset is statistically equivalent to or superior to the FS model on the dataset size indicated by “Comparable FS Size.” Labeling time for each case was computed by adding the time required to hand-label the development set to the time required to either hand-label (FS) or weakly label (DP) the training set. See Table 1 for dataset size definitions. Note that labeling times reported for full supervision are conservative, as we assume that only a single clinician contributed to reading each case.

		**Text** (Dev)			**Image** (Test)
Task	Model	F1 @ 0.5	ROC-AUC	Coverage	Median ROC-AUC
**CXR** (20 LFs)	FS	-	-	-	0.95 ± 0.005 (Med. 0.94)
	DM-GM	0.92 ± 0.006	0.95 ± 0.010	1.00	0.94 ± 0.005 (Med. 0.94)
	DM-MV	0.88 ± 0.064	0.96 ± 0.009	1.00	0.93 ± 0.007 (Med. 0.93)
	GM	0.86 ± 0.003	0.80 ± 0.005	0.88	0.88 ± 0.031 (Med. 0.89)
	MV	0.86	0.67	0.84	0.89 ± 0.019 (Med. 0.90)
**EXR** (18 LFs)	FS	-	-	-	0.91 ± 0.006 (Med. 0.90)
	DM-GM	0.83 ± 0.007	0.87 ± 0.006	1.00	0.92 ± 0.016 (Med. 0.94)
	DM-MV	0.80 ± 0.009	0.85 ± 0.011	1.00	0.87 ± 0.026 (Med. 0.88)
	GM	0.78 ± 0.001	0.84 ± 0.002	0.89	0.90 ± 0.018 (Med. 0.90)
	MV	0.82	0.80	0.77	0.88 ± 0.011 (Med. 0.88)
**HCT** (7 LFs)	FS	-	-	-	0.92 ± 0.027 (Med. 0.93)
	DM-GM	0.95 ± 0.006	1.00 ± 0.001	1.00	0.92 ± 0.037 (Med. 0.92)
	DM-MV	0.95 ± 0.004	1.00 ± 0.000	1.00	0.94 ± 0.023 (Med. 0.95)
	GM	0.96 ± 0.000	0.98 ± 0.000	1.00	0.90 ± 0.017 (Med. 0.90)
	MV	0.96	0.98	1.00	0.90 ± 0.048 (Med. 0.88)
**EEG** (11 LFs)	FS	-	-	-	0.92 ± 0.007 (Med. 0.92)
	DM-GM	0.87 ± 0.052	0.97 ± 0.010	1.00	0.84 ± 0.019 (Med. 0.85)
	DM-MV	0.81 ± 0.027	0.96 ± 0.008	1.00	0.83 ± 0.020 (Med. 0.84)
	GM	0.90 ± 0.000	0.96 ± 0.006	0.96	0.84 ± 0.023 (Med. 0.85)
	MV	0.88	0.95	0.95	0.84 ± 0.003 (Med. 0.84)

Table S4. Mean and variance of performance of different parts of the cross-modal data programming pipeline. We analyze the performance of majority vote of the labeling functions (MV), a generative model trained on these labeling functions (GM), a discriminative LSTM trained to map the raw text to the majority vote output (DM-MV), a discriminative LSTM trained to map the raw text to the generative model output (DM-GM), and hand-labeled full supervision (FS). We present F1 score at the default cutoff value of 0.5 to indicate how each model would perform as a binary classifier, and ROC-AUC to provide a more complete measurement of how well each model ranks positive and negative examples. Text model results are reported on the development (Dev) set, while downstream image model performance is reported on the held-out test (Test) set. ± represent standard deviations, and for image models we also present the median. Note that MV text model results and all coverage results are deterministic. We find that in cases where either generative model coverage or ROC-AUC is below 90%, the additional LSTM modeling step can improve text modeling performance substantially.

Supplemental Experimental Procedures

Details of Cross-Modal Data Programming Implementation for HCT

We provide a detailed description of how cross-modal data programming is implemented for the HCT application below.

We first curate and preprocess the dataset used for each application. For HCT, we create a dataset describing the binary task of intracranial hemorrhage detection by collecting 5,582 non-contrast HCT studies from our institution's Picture Archiving and Communications System (PACS) and procuring their associated text reports. We consider this problem within the *multiple instance learning* (MIL) framework, wherein an example should be considered abnormal if *any* of the individual frames contains evidence of hemorrhage.[43] We restrict our dataset to studies containing between 29 and 45 axial slices reconstructed at 5 mm axial resolution and retain the center 32 slices of each reconstruction, padding with images containing values of 0 Hounsfield Units where necessary. Clinicians then hand-label a small *development set*, which is used not as training data, but rather as an aid for tuning both clinician-provided LFs and model hyperparameters; for HCT, this required clinicians to label 170 HCT studies as positive or negative for hemorrhage.

As a second step, clinicians write LFs that for each report either provide a label or else abstain. For HCT, a single radiology fellow composed seven Python LFs over the text report based on their own experience reading and writing radiology reports, with several hours of support from a computer science graduate student. Third, we train a generative model to simultaneously learn the accuracies of all LFs. Concretely, we compute the Λ matrix by executing LF code to calculate output values for $m = 7$ LFs on $n = 4{,}000$ training examples, and then execute a single Python command in Snorkel[30] to estimate generative model parameters $\hat{\theta}$ by solving Eq. 1. Fourth, we assign a composite probabilistic label that represents an appropriately weighted combination of the LF outputs; practically, this translates into executing the trained generative model over each report. Clinicians then compare the output of the generative model to their ground truth development set labels, and repeat steps 2 - 4 until diminishing returns are observed with respect to generative model performance as evaluated against the development set. This entire procedure generally requires fewer than eight cumulative hours of clinician time per task.

We can next use our heuristic optimizer to determine whether or not to train an LSTM over the raw text to provide an augmented set of probabilistic labels. The inputs to this model would be the raw text reports, while the output targets would be the labels produced by the generative model. For HCT, the generative model labels have ROC-AUC and coverage of over 90% on the development set, so we bypass LSTM training and use the generative model labels directly as our source of weak supervision for our image model. Bypassing the LSTM training process in cases like this where we expect minimal performance improvement can save substantial amounts of computation time.

Once weak labels have been provided for a given task, a discriminative machine learning model (e.g. neural network classifier) can be trained over the raw data modality to evaluate the quantity of interest. For HCT, we define a standard attention mechanism over an 18-layer Residual network[9] encoder in PyTorch[11] that operates on every axial slice of the reconstructed tomographic image. This attention mechanism is a small neural network that dynamically learns how heavily a given slice should be weighted in the representation used by the final classification layer.[43] We then estimate optimal parameters $\hat{w}$ by approximating the solution to Eq. 2 using standard PyTorch backpropagation algorithms and a binary cross entropy loss function between the network prediction and the weak probabilistic label.

Computer Code for Cross-Modal Data Programming

We provide labeling function (LF) code for each medical imaging application and a functional demonstration of the entire cross-modal weak supervision technique on a small, public dataset at https://github.com/HazyResearch/cross-modal-ws-demo. The code provided in this demonstration exactly mirrors that used for the analysis presented in the manuscript.

Supplemental References

50. Asif, U., Roy, S., Tang, J., and Harrer, S. (2019). SeizureNet: Multi-Spectral Deep Feature Learning for Seizure Type Classification. arXiv Prepr. arXiv1903.03232.
51. Jnawali, K., Arbabshirani, M.R., Rao, N., and Patel, A.A. (2018). Deep 3D convolution neural network for CT brain hemorrhage classification. Proc. SPIE Med. Imaging 2018 Comput. Diagnosis *10575*, 105751C.
52. Roy, Y., Banville, H., Albuquerque, I., Gramfort, A., Falk, T.H., and Faubert, J. (2019). Deep learning-based electroencephalography analysis: A systematic review. J. Neural Eng. *16*.
