# Supplementary material for: Cross-Modal Data Programming Enables Rapid Medical Machine Learning
Source: Patterns (N Y). 2020 Apr 28;1(2):100019. doi: 10.1016/j.patter.2020.100019 (PMC7413132; doi:10.1016/j.patter.2020.100019)
Supplement: Document S2. Article plus Supplemental Information [file mmc2.pdf]

Article

Patterns

Cross-Modal Data Programming Enables Rapid Medical Machine Learning

Highlights

- We propose cross-modal data programming (XMDP) for machine learning (ML) in medicine
- XMDP reduces labeling costs by 96% versus hand labeling on 4 diverse medical ML tasks
- Days of XMDP and years of hand labeling often yield similarly performing models
- XMDP performance continually improves as more unlabeled data becomes available

Authors

Jared A. Dunnmon,
Alexander J. Ratner, Khaled Saab, ...,
Matthew P. Lungren, Daniel L. Rubin,
Christopher Ré

Correspondence

jdunnmon@cs.stanford.edu

In Brief

Machine learning (ML) models have achieved record-breaking performance on many tasks, but development is often blocked by a lack of large, hand-labeled training datasets for model supervision. We extend data programming—a theoretically grounded technique for supervision using cheaper, noisier labels—to train medical ML models using person-days of effort that previously required person-years of hand labeling. We find that our weakly supervised models perform similarly to their hand-labeled counterparts and that their performance improves as additional unlabeled data becomes available.

Dunnmon et al., 2020, Patterns *1*, 100019
May 8, 2020 © 2020 The Author(s).
https://doi.org/10.1016/j.patter.2020.100019

CellPress

Patterns

CellPress
OPEN ACCESS

Article

Cross-Modal Data Programming Enables Rapid Medical Machine Learning

Jared A. Dunnmon,[1,6,8,*] Alexander J. Ratner,[1,6] Khaled Saab,[2] Nishith Khandwala,[1] Matthew Markert,[3] Hersh Sagreiya,[4] Roger Goldman,[4] Christopher Lee-Messer,[3] Matthew P. Lungren,[4] Daniel L. Rubin,[4,5,7] and Christopher Ré[1,7]

[1]Department of Computer Science, Stanford University, Stanford, CA, USA
[2]Department of Electrical Engineering, Stanford University, Stanford, CA, USA
[3]Department of Neurology, Stanford University, Stanford, CA, USA
[4]Department of Radiology, Stanford University, Stanford, CA, USA
[5]Department of Biomedical Data Science, Stanford University, Stanford, CA, USA
[6]These authors contributed equally
[7]These authors contributed equally
[8]Lead Contact
*Correspondence: jdunnmon@cs.stanford.edu
https://doi.org/10.1016/j.patter.2020.100019

THE BIGGER PICTURE Machine learning can achieve record-breaking performance on many tasks, but machine learning development is often hindered by insufficient hand-labeled data for model training. This issue is particularly prohibitive in areas such as medical diagnostic analysis, where data are private and require expensive labeling by clinicians.

A promising approach to handle this bottleneck is weak supervision, where machine learning models are trained using cheaper, noisier labels. We extend a recent, theoretically grounded weak supervision paradigm—data programming—wherein subject matter expert users write labeling functions to label training data imprecisely rather than hand-labeling data points. We show that our approach allows us to train machine learning models using person-days of effort that previously required person-years of hand labeling. Our methods could enable researchers and practitioners to leverage machine learning models over high-dimensional data (e.g., images, time series) even when labeled training sets are unavailable.

1 2 3 4 5 **Development/Pre-production:** Data science output has been rolled out/validated across multiple domains/problems

SUMMARY

A major bottleneck in developing clinically impactful machine learning models is a lack of labeled training data for model supervision. Thus, medical researchers increasingly turn to weaker, noisier sources of supervision, such as leveraging extractions from unstructured text reports to supervise image classification. A key challenge in weak supervision is combining sources of information that may differ in quality and have correlated errors. Recently, a statistical theory of weak supervision called data programming has shown promise in addressing this challenge. Data programming now underpins many deployed machine-learning systems in the technology industry, even for critical applications. We propose a new technique for applying data programming to the problem of cross-modal weak supervision in medicine, wherein weak labels derived from an auxiliary modality (e.g., text) are used to train models over a different target modality (e.g., images). We evaluate our approach on diverse clinical tasks via direct comparison to institution-scale, hand-labeled datasets. We find that our supervision technique increases model performance by up to 6 points area under the receiver operating characteristic curve (ROC-AUC) over baseline methods by improving both coverage and quality of the weak labels. Our approach yields models that on average perform within 1.75 points ROC-AUC of those supervised with physician-years of hand labeling and outperform those supervised with physician-months of hand labeling by 10.25 points ROC-AUC, while using only person-days of developer time and clinician work—a time saving of 96%. Our results suggest that modern weak supervision techniques such as data programming may enable more rapid development and deployment of clinically useful machine-learning models.

This is an open access article under the CC BY license (http://creativecommons.org/licenses/by/4.0/).

INTRODUCTION

Modern machine learning approaches have achieved impressive empirical successes on diverse clinical tasks that include predicting cancer prognosis from digital pathology,[1,2] classifying skin lesions from dermatoscopy,[3] characterizing retinopathy from fundus photographs,[4] detecting intracranial hemorrhage through computed tomography (CT),[5,6] and performing automated interpretation of chest radiographs (CXRs).[7,8] These applications typically use standard neural network architectures[9] that are supported in professionally maintained open-source frameworks and can be downloaded within minutes from online repositories.[10,11] This trend suggests that model design is no longer a major barrier to entry in medical machine learning. However, each of these application successes was predicated on the existence of massive hand-labeled training datasets that often require physician-years of labeling time to create, cost hundreds of thousands of dollars per task, and are not robust to changes in the data distribution or labeling schema.[4,12] Reducing the dependence of clinically impactful machine learning models on costly hand-labeled data would enable more rapid deployment to provide value in medical practice.[12]

Reliance on hand-labeled datasets is not a challenge unique to medicine. Similar problems in both industry and academia have recently been addressed using advances in weak supervision, where training data can be rapidly labeled in noisier, higher-level, often programmatic ways rather than manually by experts. The statistical intuition behind these techniques is that a larger volume of noisier training data can sometimes be more effective than a smaller amount of hand-labeled data. Similar ideas have historically driven a rich set of work on topics such as template-based systems for knowledge-base construction,[13] pattern-oriented bootstrapping for relation extraction,[14] and co-training techniques for leveraging unlabeled data.[15] Weak supervision has already had a substantial impact on how machine learning models are built in the technology industry, where these methods have been used to build models for critical applications that perform better than their hand-labeled counterparts while using fewer labeling resources.[16–22] Our work assesses whether the benefits of these same weak supervision techniques can be translated from industrial use cases into clinical impact by reducing the amount of hand-labeled data required to train clinically useful machine learning models.

A common difficulty in applying weak supervision methods in medicine is that providing even noisy labels can be difficult if only raw image or sensor data are available. In this context, a promising approach is one we call cross-modal weak supervision, where noisy labels are programmatically extracted from an auxiliary modality (e.g., the unstructured text reports accompanying an imaging study) and then used to train a model over a target modality (e.g., the medical images). At test time, we would like to obtain a prediction from the target modality but usually do not have access to the auxiliary modality—for instance, the text report has not yet been written when the image is first acquired. Recent research in medical imaging has used cross-modal weak supervision by applying natural language-processing techniques to clinical reports accompanying the images of interest.[5,8,23–26] Such approaches are compelling because important medical information that could be used for labeling is often encoded in unstructured forms (e.g., free text) that are not suitable for supervising machine learning models.

The key technical challenge in applying such weak supervision techniques in practice is combining sources of information that may overlap, conflict, and be arbitrarily correlated in order to provide accurate training labels for a machine learning algorithm. While a variety of previous cross-modal weak supervision approaches have shown promise in medical applications, studies performed to date have generated weak labels from text in ad hoc, application-specific ways. Specific strategies reported in the medical literature range from combining pre-existing disease tagging tools[27,28] with handcrafted negation detection rules to provide labels for CXR classification[8,23] to ensembling large numbers of distinct regressors that predict specific medical concepts to provide labels for cranial abnormality detection from CT.[5] None of these application-specific cross-modal weak supervision approaches are supported by theoretical analysis that characterizes how well the resultant models are expected to perform. Furthermore, because large, hand-labeled datasets are generally unavailable when cross-modal weak supervision is used in medicine, empirical comparison of these models' performance with those trained using hand labels has not yet been possible. Because neither theoretical nor empirical analysis of how cross-modal weak supervision methods perform across diverse clinical use cases has been conducted, it remains difficult for practitioners to confidently deploy these models to improve patient care.

In this work, we propose a theoretically grounded, application-agnostic approach to cross-modal weak supervision and apply this technique to diverse, institution-scale hand-labeled clinical datasets to perform the first comprehensive study of cross-modal weak supervision in medicine. Such a study allows us to directly assess both the merits of our proposed approach and the broader translational potential of models trained using cross-modal weak supervision across multiple clinical use cases. We directly compare our cross-modal weak supervision technique with hand labeling along three principal axes: absolute model performance, model performance improvement with additional training data, and the type and amount of labeling resources required. We further evaluate how our supervision approach improves model performance via a series of ablation studies. We make four distinct contributions in the course of this work.

First, we propose a single, uniform approach to cross-modal weak supervision that is theoretically grounded, is applicable across diverse medical-use cases, and exhibits high levels of performance when compared with hand labeling. We base our technique on data programming, an application-agnostic weak supervision technique recently developed and theoretically analyzed by Ratner et al.[29–31] Data programming uses a generative modeling step to create weak training labels by combining unlabeled data with heuristics provided by domain experts that may overlap, conflict, and be arbitrarily correlated. Data programming has also recently been shown to consistently outperform simple heuristic ensembling strategies such as majority vote across use cases spanning academic natural language processing (NLP) benchmarks, biomedical text classification tasks, and core products in the technology industry, making it a compelling method on which to base our approach.[16,30] To

CellPress
OPEN ACCESS

handle cross-modal applications where it is challenging to enumerate sets of heuristics that provide useful supervision signal on every example in the dataset, we propose the use of a learned neural network to improve both the coverage and quality of the weak labels emitted by the base data-programming model. Our work represents the first extension of data programming, and the associated open-source Snorkel software,[30] to diverse, clinically important imaging and monitoring tasks in the cross-modal setting. We refer to the specific cross-modal weak supervision approach we propose here as "cross-modal data programming."

Second, to support an analysis of cross-modal weak supervision that generalizes across applications, we curate four diverse medical datasets in applications spanning CXR and knee extremity radiograph (EXR) triage, intracranial hemorrhage identification on head CT (HCT), and seizure onset detection on electroencephalography (EEG), each of which has important clinical implications previously described in the literature.[5,7,32,33] In contrast to previous work, we evaluate our cross-modal weak supervision technique by directly comparing performance and resource requirements for models trained using our approach with those trained using hand labels provided over the exact same data. We view the curation of the diverse institution-scale datasets required to perform these comparisons—each of which contains raw images or signals, associated clinical reports, and clinician-provided hand labels—as a major contribution of this work. This type of comparative analysis is critical to evaluating the translational potential of models trained using cross-modal weak supervision, but has not yet been performed because large, hand-labeled datasets with associated text-image pairs are rarely available and because previous work does not report the amount of domain expert time and resources required to perform cross-modal weak supervision (e.g., training an NLP model for label extraction).

Third, we present empirical evidence that models for clinically important applications that have been trained using cross-modal weak supervision can perform similarly to those trained using large hand-labeled datasets while providing several practical advantages. Across applications, we find that models weakly supervised using cross-modal data programming over all available data outperform models trained with physician-months of hand-labeled data by an average of 10.25 points of area under the receiver-operating characteristic curve (ROC-AUC). We additionally find that models trained using cross-modal data programming over all available data achieve on average within 1.75 points ROC-AUC of models trained using hand labels over all available data, which required physician-years to obtain. These results suggest that cross-modal data programming can yield models with similar clinical utility to those trained with hand labels. We further observe that the performance of models trained using cross-modal data programming and hand labeling both improve with additional training data, and often at similar rates. Not only is this behavior consistent with theoretical predictions, but it also emphasizes that weakly supervised model performance can be improved by providing more unlabeled data with no additional clinician effort. Finally, we find that cross-modal data programming required less than 8 h of clinician time per application, which was largely spent defining simple pattern-matching or ontology-lookup heuristics over clinical text reports. In our experiments, models trained with cross-modal data programming achieved statistical equivalence to those trained using hand labels while providing an average 96% saving in labeling time. Our results provide evidence that cross-modal data programming can lower a substantial barrier to machine-learning model development in medicine by reducing the labeling time required from domain experts by orders of magnitude while maintaining high levels of performance.

Fourth, our ablation studies demonstrate that modeling steps within cross-modal data programming result in target modality models that perform up to 6 points ROC-AUC better than those trained using majority vote of clinician-provided heuristics, which serves as a proxy for common rule-based approaches to cross-modal weak supervision. Our analysis suggests that these performance improvements are caused by increases in both the coverage and quality of the weak labels with respect to the majority-vote baseline, and that our proposed neural network approach emits weak labels that yield a statistically significant performance improvement for target modality models. We find that these gains are more pronounced on screening tasks such as radiograph triage and marginal on more targeted detection tasks such as hemorrhage or seizure detection on which clinician-provided heuristics are both highly accurate and cover most of the dataset. Thus, we not only demonstrate that the specific cross-modal weak supervision technique proposed in this work can improve performance over strong baselines but also identify the clinical contexts in which each component of our approach may be particularly useful.

We first describe technical details of our cross-modal data programming approach. We then provide an overview of the institution-scale, hand-labeled datasets curated for this study and outline our process for applying cross-modal data programming to these use cases using a consistent procedure. We next compare models trained using cross-modal data programming against their hand-labeled counterparts, assessing absolute levels of performance, performance scaling with the amount of training data, and required labeling resources. Finally, we assess the empirical utility of each component of our cross-modal data programming modeling approach via a series of ablation studies and discuss important implications of our results for translational medicine.

RESULTS

Overview of Cross-Modal Data Programming

The data programming method of Ratner et al.[29] handles the label noise inherent in weak supervision by automatically estimating the accuracies and correlations of different labeling heuristics in an unsupervised manner, reweighting and combining their outputs, and finally producing a set of probabilistic training labels that can be used to supervise a deep learning model. We build on these ideas by proposing cross-modal data programming.

In cross-modal data programming (Figure 1), users such as clinicians provide two basic inputs: first, unlabeled cross-modal data points, which are represented as target-auxiliary modality pairs $(x_t^{(i)}, x_a^{(i)}) \in \mathcal{X}_t \times \mathcal{X}_a$ (e.g., an imaging study and the accompanying text report); and second, a set of labeling functions (LFs), $\{\lambda_j\}$, which are user-defined functions (e.g., pattern-matching rules, existing classifiers) that take in an auxiliary modality data point $x_a^{(i)}$ as input and either output a label, $\lambda_j^{(i)} \in \mathcal{Y}$, or abstain ($\lambda_j^{(i)} = 0$). Here, we consider the binary setting $\mathcal{Y} = \{-1, 1\}$,

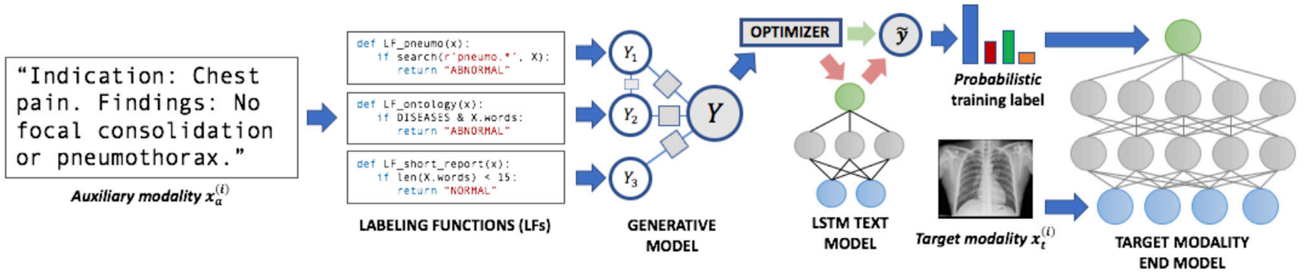

Figure 1. A Cross-Modal Data Programming Pipeline for Rapidly Training Medical Classifiers

A clinician first writes several labeling functions (LFs), which are Python functions that express pattern-based rules or other heuristics over the auxiliary modality (e.g., text report). Here, for instance, the function *LF_pneumo* would label a text report as "abnormal" if the report contains a word with the prefix "pneumo"; note that these LFs may overlap, conflict, and be arbitrarily correlated with each other. During the offline model training phase, LFs are applied to unstructured clinician reports and combined to generate probabilistic (confidence-weighted) training labels for a classifier defined over the target modality (e.g., radiograph). A discriminative text model such as a long short-term memory (LSTM) network can then be trained to map the raw text to the generative model output. Our optimizer uses a simple heuristic to determine whether computation can be saved by training the target modality end model directly on probabilistic labels emitted from the generative model or if end model performance can be improved by using probabilistic labels emitted from the trained LSTM. At test time, the end model receives only the target modality as input and returns predictions.

corresponding to, for example, "normal" and "abnormal," and make the assumption that the majority of these LFs will be more accurate than random chance.[29] In practice, users often create a small, hand-labeled development set (e.g., several hundred examples) to assist in LF development and tuning.

Given m such LFs, we apply them to the unlabeled auxiliary modality data points $\{x_a^{(i)}\}_{i=1,\ldots,n}$ to generate a matrix $\Lambda \in \mathbb{R}^{n\times m}$ of noisy labels, where the non-zero elements in each row Λ_i represent the training labels generated by the LFs for point i. In general, these labels may overlap, conflict, and be arbitrarily correlated. The goal of data programming is to reweight and combine them to generate a single probabilistic (i.e., confidence-weighted) label $\tilde{y}^{(i)}$. To do this, we use an unsupervised generative modeling procedure to learn the LF accuracies θ that best explain the observed labeling pattern via minimization of the negative log marginal likelihood[29]

$$\widehat{\theta} = \text{argmin}_\theta \left(-\log \sum_Y p_\theta(Y, \Lambda) \right), \qquad \text{(Equation 1)}$$

where p_θ is an exponential family generative model and Y is the unobserved true label vector over which we marginalize.

The result of this first modeling stage is a set of probabilistic or confidence-weighted training labels $\tilde{y}^{(i)}$. In the simplest case, these labels are used to train a weakly supervised discriminative model f^t (e.g., a neural network) over the target modality t using a noise-aware variant of the loss function l,[29] which takes into account the uncertainty of the probabilistic training labels:

$$\widehat{w}^t = \text{argmin}_{w^t} \sum_{i=1}^{n} \mathbb{E}_{\tilde{y}^{(i)} \sim p_{\widehat{\theta}}(\cdot|\Lambda_i)} \left[l\left(f^t\left(x_t^{(i)}; w^t\right), \tilde{y}^{(i)}\right)\right]. \qquad \text{(Equation 2)}$$

The resulting model $f^t(\cdot; \widehat{w}^t)$—represented by the estimated parameters $\widehat{w}^t$—can then be applied to the target modality alone; for example, to classify CXRs by triage priority before human interpretation. Importantly, data programming relies on recent statistical learning theory,[31] which guarantees that, under certain basic statistical assumptions, the estimation error of the learned LF accuracies and correlation parameters (Equation 1) and the test error of the discriminative model (Equation 2) will both be upper bounded by $O\left(n^{-\frac{1}{2}}\right)$. That is, as the size n of the unlabeled dataset increases with the LFs held constant—i.e., with no additional supervision signal—we expect to observe improved performance at the same asymptotic rate as in traditional supervised approaches when adding more hand-labeled samples. This result, recently derived by Ratner et al.,[31] provides a theoretical framework for understanding a broad spectrum of cross-modal weak supervision methods and how they can leverage both clinician domain expertise and available unlabeled data.

The cross-modal setting presents an opportunity to improve this basic data-programming pipeline via an additional modeling step that we propose here. Specifically, the probabilistic training labels $\tilde{y}^{(i)}$ produced by the generative model do not always provide full coverage, as all LFs may abstain on any given example.[30] We mitigate the effects of incomplete coverage by training an intermediate discriminative model f^a, parameterized by weights w^a, that predicts the generative model output directly from the raw auxiliary modality a. Concretely, we can create an augmented set of probabilistic labels $\tilde{y}_a^{(i)}$ by estimating $\widehat{w}^a$ as

$$\widehat{w}^a = \text{argmin}_{w^a} \sum_{i=1}^{n} \mathbb{E}_{\tilde{y}^{(i)} \sim p_{\widehat{\theta}}(\cdot|\Lambda_i)} \left[l\left(f^a\left(x_a^{(i)}; w^a\right), \tilde{y}^{(i)}\right)\right] \qquad \text{(Equation 3)}$$

and subsequently evaluating f^a over all training points,

$$\tilde{y}_a^{(i)} = f^a\left(x_a^{(i)}; \widehat{w}^a\right). \qquad \text{(Equation 4)}$$

In this way, we can produce a set of training labels that approximately retains the favorable statistical properties described above but provides useful labels for every element of the training set. The target modality model is then trained exactly as in Equation 2, but using $\tilde{y}_a^{(i)}$ instead of $\tilde{y}^{(i)}$:

$$\widehat{w}^t = \text{argmin}_{w^t} \sum_{i=1}^{n} \mathbb{E}_{\tilde{y}_a^{(i)}} \left[l\left(f^t\left(x_t^{(i)}; w^t\right), \tilde{y}_a^{(i)}\right)\right]. \qquad \text{(Equation 5)}$$

CellPress
OPEN ACCESS

As illustrated in Figure 1, we implement this idea on paired text-image datasets by training a standard long short-term memory (LSTM) network over text reports (the auxiliary modality) using probabilistic labels $\tilde{y}^{(i)}$ generated by combining our LFs within the generative modeling step.[34] We use a simple heuristic optimizer to decide whether to use the generative model probabilistic labels $\tilde{y}^{(i)}$ directly or if performance could be improved by using an augmented set of probabilistic labels $\tilde{y}_a^{(i)}$ emitted from the trained LSTM model. Specifically, we train the LSTM only if either generative model coverage or ROC-AUC on the development set is less than 90%. Bypassing the LSTM training step in cases where we would expect end model performance to be equivalent using the generative model labels can save substantial computation. We explicitly assess the effect of each step in this cross-modal data programming pipeline as part of our experimental analysis.

We propose using cross-modal data programming as an exemplar technique for evaluating the efficacy of training medical machine learning models with cross-modal weak supervision for several compelling reasons. First, data programming naturally extends and improves upon common rule-based methods by using unsupervised statistical modeling techniques to automatically denoise potentially overlapping and conflicting heuristic functions in a way that provides more accurate, probabilistic training labels to deep learning models.[29] This allows us to directly compare with a majority vote over our LFs as a proxy for existing, application-specific cross-modal weak supervision methods that commonly use rule-based procedures. Second, theoretical analysis suggests that this approach should yield continual performance improvement with increasing amounts of unlabeled data.[31] Third, the generative modeling formulation allows us to easily add an intermediate neural network model that can improve the coverage of our weak labels while approximately retaining these theoretical guarantees. Finally, data programming is well suited to evaluating the resource requirements of cross-modal weak supervision relative to hand labeling because it is supported by Snorkel,[30] a well-validated, open-source software system that enables cross-modal weak supervision across different applications in a manner that is both accessible to clinicians and amenable to detailed quantitative analysis.

Datasets and Experimental Procedure

We use the theoretically grounded cross-modal data programming approach described above to assess the performance of cross-modal weak supervision in four real-world medical applications (Figure 2) spanning CXR triage (two-dimensional [2D] image classification), EXR series triage (2D image series classification), intracranial hemorrhage detection on CT (three-dimensional [3D] volumetric image classification), and seizure-onset detection on EEG (19-channel time-series classification). We use a standard deep neural network architecture for the target modality in each application, as described in Table 1. Fixing the model class and training procedure for each application allows us to assess the effects of different supervision approaches with all other variables held constant. To provide a rigorous comparison between cross-modal data programming and hand-labeled data in our experiments, we curate a large hand-labeled dataset for each application comprising raw data, associated reports, and clinician-provided labels. Each of these datasets represents physician-years of hand labeling. Using these data resources, we can assess how closely the performance of models trained using cross-modal data programming can come to matching their fully supervised equivalents in the context of real clinical data.

For each application, we apply the cross-modal data-programming approach described above, which is implemented as an extension to the Snorkel[30] software package: a clinician first writes LFs over the text reports using a small, hand-labeled development set for LF tuning; we then use Snorkel to generate a final set of probabilistic training labels, using the intermediate LSTM text model if generative model coverage or ROC-AUC is below 90% on the development set; and we finally train a discriminative model over the target data modality. We report implementation details for each application in Experimental Procedures and present an in-depth walkthrough of how cross-modal data programming was applied to the HCT application in Supplemental Experimental Procedures. Complete code for all LFs is provided in Supplemental Experimental Procedures, along with a Jupyter notebook containing a step-by-step tutorial on how to apply cross-modal data programming to a small, publicly available dataset.[38]

We use this cross-modal data-programming approach to empirically assess the hypothesis that cross-modal weak supervision methods can reduce the amount of labeling resources required to build useful machine-learning models across diverse clinical settings. We compare the absolute performance of cross-modal data programming with hand-labeled supervision in Figure 3, provide empirical scaling results compared with hand-labeled supervision in Figure 4, analyze the amount of labeling resources required relative to hand-labeled supervision in Figure 5, and assess the performance effects of each part of the cross-modal data programming pipeline in Figure 6.

Weakly Supervised Performance Matches or Exceeds Fully Supervised Performance

We first assess the degree to which models trained with cross-modal data programming and standard model architectures (see Experimental Procedures) can approach the performance of those trained using hand-labeled datasets. From a translational standpoint, this experiment evaluates whether cross-modal weak supervision can allow large hand-labeled training sets to be replaced with flexible and high-level programmatic supervision specified by clinicians. In Figure 3, we compare cross-modal data programming with hand-labeled full supervision with Large and Medium hand-labeled datasets (see Table 1 for dataset size definitions). Note that data programming refers to either the LSTM or generative model output, as determined by the optimizer step; we analyze cases in which the LSTM versus the generative model should be used in a later section. We find that across applications, median models (i.e., models achieving median ROC-AUC across five random seeds) trained using cross-modal data programming are able to perform within an average of 1.75 points ROC-AUC of those trained with Large hand-labeled datasets while outperforming those trained using Medium hand-labeled datasets by an average of 10.25 points ROC-AUC. Median weakly supervised models for CXR and HCT are not statistically different from median models trained using large hand-labeled datasets (DeLong $p > 0.38$),[41] the median weakly supervised EXR model performs several points

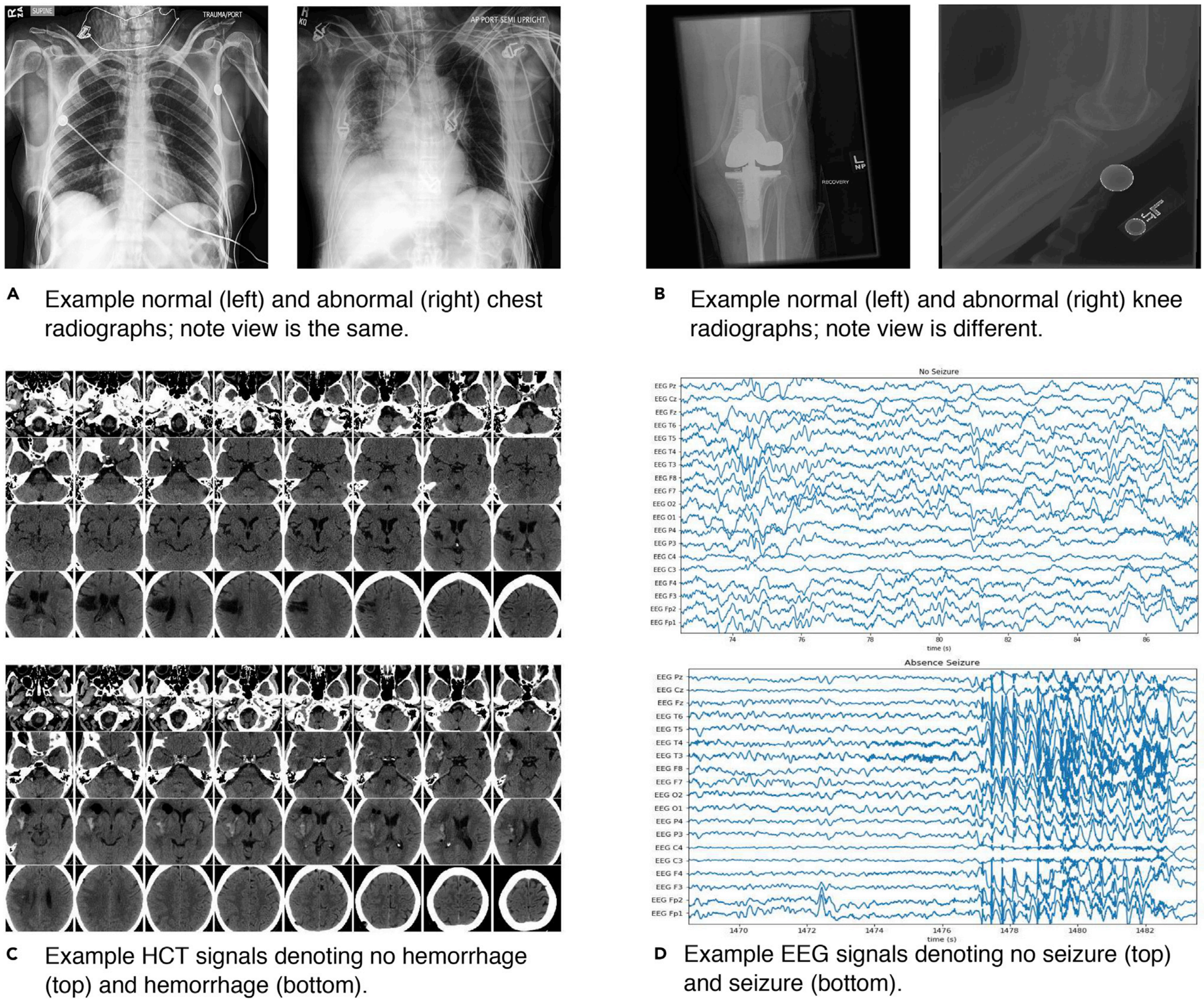

A Example normal (left) and abnormal (right) chest radiographs; note view is the same.

B Example normal (left) and abnormal (right) knee radiographs; note view is different.

C Example HCT signals denoting no hemorrhage (top) and hemorrhage (bottom).

D Example EEG signals denoting no seizure (top) and seizure (bottom).

Figure 2. Example Target Modality Data for Four Clinical Applications

Single 2D chest radiographs (A), examples of knee extremity radiographs drawn from 2D radiograph series (B), 32 slices from 3D head CT scans (HCT) with and without hemorrhage (C), and 19-channel electroencephalography (EEG) signals with and without evidence of seizure onset (D). Note that while these applications are fundamentally different in both dimensionality and modeling requirements (see Table 1), deep machine learning models supporting each can be rapidly trained using cross-modal data programming.

ROC-AUC higher than the median model trained using the Large hand-labeled dataset (DeLong $p < 0.05$), and median weakly supervised EEG models are not statistically different from median models trained using Medium hand-labeled datasets (DeLong $p > 0.25$). Exact p values for these comparisons are provided in Table S1. We view the impressive empirical performance of the weakly supervised models as compared with the fully supervised ones as a notably positive outcome of our study, and in turn view the annotation and curation effort required to perform this comparison between weakly and fully supervised models as a major contribution of this work. Furthermore, the fact that our fully supervised results compare favorably with recently published work on each problem (Table S2) reflects encouragingly on the potential clinical utility of analogous weakly supervised models.[6,7,32,42]

Cross-Modal Data-Programming Models Improve with More Unlabeled Data

A major trend in the modern clinical world is that large amounts of digitized medical data are increasingly accessible, but important information is often encoded in unstructured forms (e.g., free text) that are not suitable for supervising machine-learning models. We therefore evaluate whether cross-modal weak supervision can harness these growing unstructured data resources without requiring additional clinician effort. Specifically, we assess the performance of the target modality model when additional unlabeled data are weakly labeled using cross-modal data programming, but without any modification of the fixed set of clinician-authored LFs. In Figure 4, we observe favorable performance scaling across all applications studied. In particular, the CXR results of Figure 4A demonstrate that the ROC-AUC

Table 1. Description of Data Type, Classification Task, Training-Set Sizes, and Neural Network Architectures for Each Application Studied

	CXR	EXR	HCT	EEG
Data type	single 2D radiograph	multiple 2D radiograph views	3D CT reconstruction	19-channel EEG time series
Classification task	normal	normal	hemorrhage	seizure onset
	abnormal	abnormal	no hemorrhage	no seizure onset
Anatomy	chest	knee	head	head
Train set size (Large/Medium)	50,000	30,000	4,000	30,000
	5,000	3,000	400	3,000
Train Set Size (Literature)	20,000[7]	40,561[32]	904[6]	23,218[33]
Network architecture	2D ResNet-18[9]	patient-averaged 2D ResNet-50[9]	3D MIL + ResNet-18 + Attention[35]	1D Inception DenseNet[36]

We apply cross-modal data programming to four different data types: 2D single chest radiographs (CXR), 2D extremity radiograph series (EXR), 3D reconstructions of computed tomography of the head (HCT), and 19-channel electroencephalography (EEG) time series. We use two different dataset sizes in this work: the full labeled dataset (large) of a size that might be available for an institutional study (i.e., physician-years of hand labeling) and a 10% subsample of the entire dataset (medium) of a size that might be reasonably achievable by a single research group (i.e., physician-months of hand labeling). For context, we present the size of comparable datasets used to train high-performance models in the literature. Finally, we list the different standard model architectures used. While each image model uses a residual network encoder,[9] architectures vary from a simple single-image network (CXR) to a mean across multiple image views (EXR) to a dynamically weighted attention mechanism that combines image encodings for each axial slice of a volumetric image (HCT). For EEG time series, an architecture combining the best attributes of the Residual and Densely Connected[37] networks for 1D applications is used, in which each channel is encoded separately and a fully connected layer is used to combine features extracted from each (see Experimental Procedures).

score of the weakly supervised model improves with additional unlabeled data at a similar rate to that at which the fully supervised model improves with additional hand-labeled data, as suggested by recent theoretical analysis.[29,31] Figure 4B, describing EXR triage, shows analogous scaling trends, but the initial improvement in performance of the weakly supervised model upon adding more data points appears far more rapid. We speculate that this is due to the combination of weak supervision with the relatively simple multiple-instance labeling (MIL) modeling approach used for this application (i.e., mean across radiographs), which causes the model to perform particularly poorly at small sample sizes. These scaling trends also hold for more complex modalities in HCT and EEG in Figures 4C and 4D. For HCT, we observe that the weakly supervised and fully supervised results in this case are extremely similar at all dataset sizes; this behavior likely results from well-engineered LFs that yield particularly accurate labels over the text reports. While the gap between the weakly and fully supervised EEG results is larger than those in other applications, this is not unexpected given the known difficulty of the problem and the fact that we work with pediatric signals, which generally exhibit high levels of patient-to-patient variation.[42] Across applications, we find that mean performance of the weakly supervised models improves substantially with additional examples and observe that model variance across training runs with different random seeds often decreases as training-set size increases. Thus, our scaling results suggest that the performance of machine learning models supervised using weak supervision techniques such as cross-modal data programming can be continually improved by harnessing additional unlabeled data, which requires no extra labeling effort from clinicians.

Cross-Modal Data Programming Requires Simple and Minimal Clinician Input

We also analyze the time and input complexity that were required of clinicians in cross-modal data programming relative to hand-labeling data. On average, 14 LFs comprising on average six lines of code each were developed per application, using a mix of general text patterns (e.g., identifying non-medical words indicating normalcy, equivocation), medical text patterns (e.g., identifying specific terms or overlaps with medical ontologies), and structural heuristics (e.g., shorter reports tend to describe normal cases), as described in Figure 5A. Importantly, between the CXR and EXR applications, six LFs were directly reused, demonstrating the ability of weak supervision methods such as data programming to amortize clinician effort across different modeling tasks. While composing LFs took less than a single physician-day per application, hand-labeling training sets required physician-months to physician-years, as shown in Figure 5B. We observe that weakly supervised models are able to attain statistically equivalent performance to supervised models while using on average 4% of the amount of physician-hours for labeling; exact values are reported in Table S3, and relative time-performance values are presented graphically in Figure 5C. Thus, we see not only that cross-modal data programming can support models that achieve high levels of performance, but also that it can do so while requiring orders-of-magnitude less time spent on the labeling process and directly incorporating relevant domain knowledge.

Cross-Modal Data Programming Can Improve Cross-Modal Weak Supervision Performance

A final hypothesis of this work is that modeling steps within cross-modal data programming are important to providing performance benefits for the target modality model. We evaluate this hypothesis in two steps. First, we assess the quality of weak labels emitted from each step in the modeling pipeline of Figure 1 by comparing the coverage and ROC-AUC of each of the following text models: an unweighted majority vote of the LFs (MV), the data-programming generative model (GM), an LSTM discriminative text model trained on MV (DM-MV), and an LSTM discriminative text model trained on GM (DM-

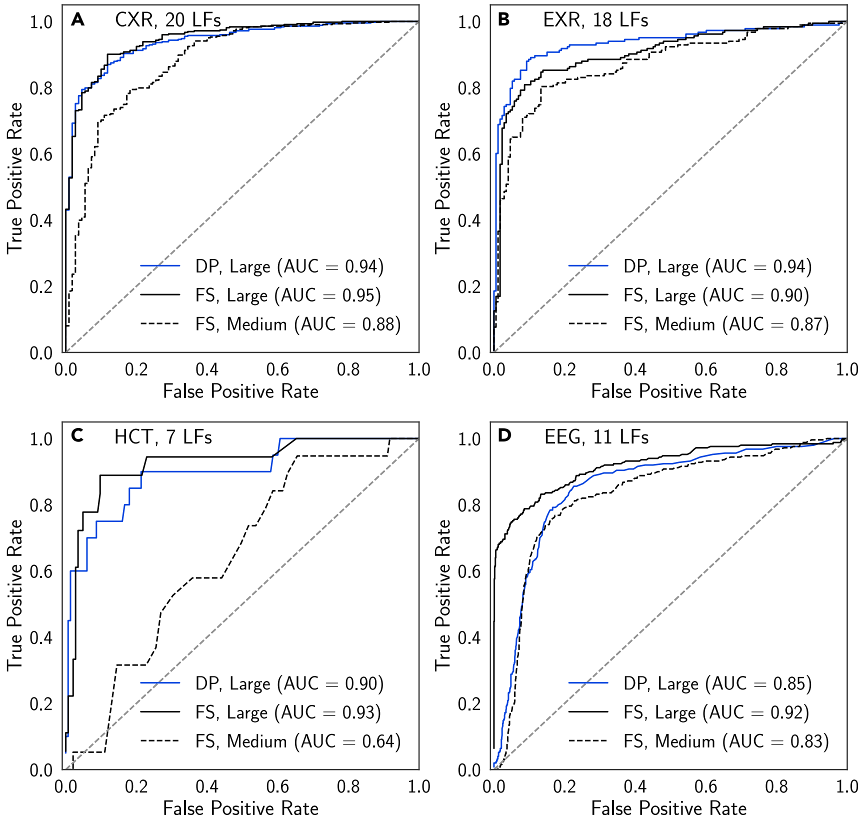

Figure 3. ROC Curves for Models Trained Using Full Hand-Labeled Supervision and Cross-Modal Data Programming

Results are presented for chest radiographs (CXR) (A), extremity radiographs (EXR) (B), head CT (HCT) (C), and electroencephalography (EEG) (D). DP, cross-modal data programming; FS, full hand-labeled supervision. Each curve shown is that attaining the median ROC-AUC score on the test set over runs using five different random seeds (see Experimental Procedures). Models trained using cross-modal data programming exhibit performance levels that meet or exceed those of models trained with Medium fully supervised datasets (i.e., physician-months of labeling time) and approach or exceed those of models trained with Large fully supervised datasets (i.e., physician-years of labeling time). See Table 1 for additional details of dataset sizes.

GM). As a proxy for common heuristic approaches to cross-modal weak supervision, MV represents a strong baseline for GM.[30] Comparing DM-GM with GM indicates the degree to which our proposed LSTM step improves performance over the GM output, and comparing DM-GM with DM-MV indicates how important the generative modeling step is to achieving the improvements observed from the LSTM. We expect that the GM will improve label quality (as measured by ROC-AUC) by resolving label overlaps and noise, while the discriminative LSTM model may both improve label coverage and further increase probabilistic label quality. Second, we compare the performance of target modality models trained on labels generated using each of these approaches with that attained using hand-labeled full supervision. This procedure allows us to empirically evaluate the effect of both the generative and discriminative text modeling steps on target modality model performance.

Performance of each text model on the development set is presented in Figure 6 for each application, while exact values—including additional metrics such as precision, recall, and F1 score—are provided in Table S4. We first find that our GM text model ROC-AUC is strictly superior to that of MV and that it provides larger improvements—on average 8.5 points ROC-AUC—on CXR and EXR applications where we have more LFs that can overlap and conflict. We also find that both DM-GM and DM-MV provide improvements in coverage and strictly non-inferior performance with respect to the LF-based MV and GM approaches for both CXR and EXR applications. On HCT and EEG, the clinician-provided rules are both full coverage and highly performant, limiting the additional utility of the intermediate LSTM text model. These results are in line with our heuristic optimizer, which would train an LSTM model only if either ROC-AUC or coverage of the GM labels is less than 90%. Thus, in the results of Figures 3, 4, and 5, we have used models trained on DM-GM labels for CXR and EXR ($\tilde{y}_a$) and models trained on GM labels ($\tilde{y}$) for HCT and EEG. This procedure allows us to save substantial computation—between GPU-hours and GPU-days depending on the application and available hardware—while producing median target modality models for HCT (DeLong p = 0.72) and EEG (DeLong p = 0.75) that are not statistically different than those trained using labels emitted from the LSTM.

In Figure 6, we also present the performance of target modality models trained on labels from each label source on Large data-set sizes; numerical mean, median, and standard deviation for each case are recorded in the rightmost column of Table S4. The effect of the different text modeling approaches on target modality model performance varies by application. We perform statistical comparisons between the ROC-AUC values of the median model for each case so that outliers do not affect our overall performance analysis. On CXR, we see that the median target modality models trained on DM-GM and DM-MV labels are only 1 point ROC-AUC apart, but are on average 4 points ROC-AUC higher than those trained on GM (DeLong p = 0.0012) and MV (DeLong p = 0.0020) labels, respectively. These general trends correlate directly with those seen in the text modeling results, where the LSTM models consistently and substantially outperform the LF-based models. Thus, on this application, our results suggest that gains in performance from additional modeling stem mostly from the improvements in ROC-AUC and coverage resultant from the intermediate LSTM step we propose in this work. We observe a slightly different trend on the EXR dataset. In this case, MV and DM-MV models perform similarly, while we see a 2-point ROC-AUC median performance improvement when using the GM labels (DeLong p = 0.15) and a further 4-point ROC-AUC increase (DeLong p =

CellPress
OPEN ACCESS

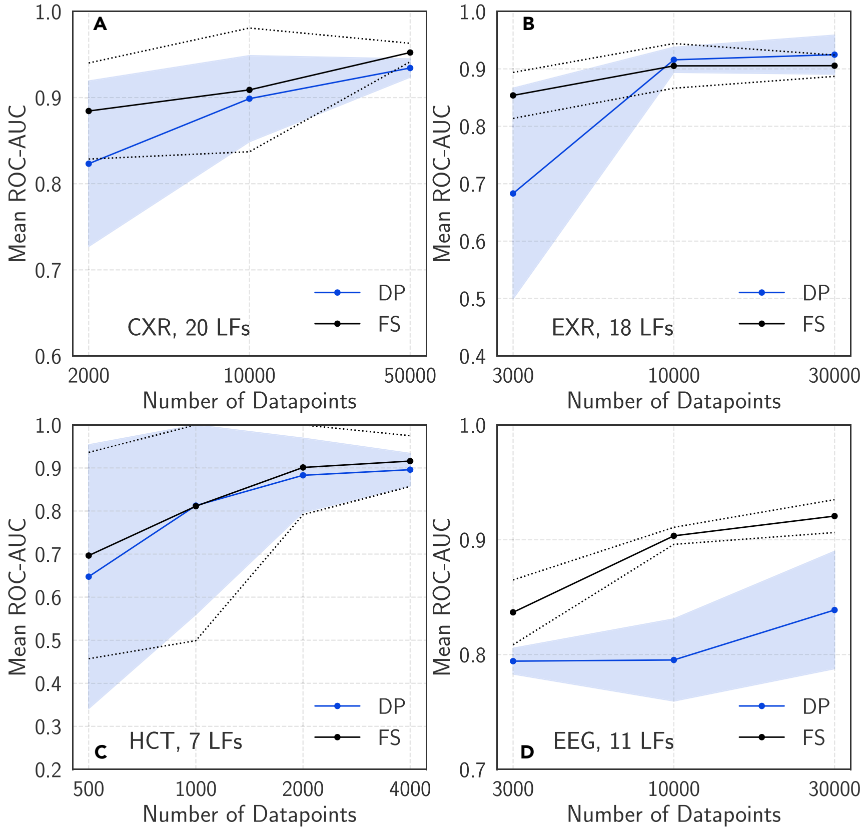

Figure 4. Mean Neural Network ROC-AUC Score versus Dataset Size Using Full Supervision and Cross-Modal Data Programming
Results are presented for chest radiographs (CXR) (A), extremity radiographs (EXR) (B), head CT (HCT) (C), and electroencephalography (EEG) (D). DP, cross-modal data programming; FS, full hand-labeled supervision. In each case, the performance of models trained using cross-modal data programming improve as additional unlabeled data are added, and in several cases exhibit scaling properties very similar to those of the fully supervised model as additional labeled data are added. Error bars (dashed lines for FS, shaded region for DP) represent 95% confidence intervals from five training runs with different random seeds.

0.0064) when using DM-GM labels. These relative results suggest that for EXR, the additional coverage provided by the discriminative model was only helpful when the LSTM was able to learn from the higher-quality GM labels. Finally, HCT and EEG represent use cases in which the LFs provide both high coverage and high performance when combined via majority vote. We observe that models trained using DM-GM labels in Figures 6C and 6D perform at levels similar to those trained using GM model labels, as our heuristic optimizer predicts.

DISCUSSION

In this work, we have performed the first comprehensive study of cross-modal weak supervision in medicine and have empirically assessed the hypothesis that cross-modal weak supervision methods can reduce the amount of labeling resources required to build useful machine learning models across diverse clinical settings. We have analyzed this class of techniques by proposing the cross-modal data programming approach, applying it to clinically relevant applications spanning radiography, CT, and EEG, and performing a direct comparison with large-scale hand labeling. Our results show that cross-modal weak supervision can enable clinicians to rapidly generate large training sets in a matter of hours or days that match or exceed the performance of large, hand-labeled training sets that took months or years of physician time to annotate. We have further provided evidence that weak supervision approaches such as cross-modal data programming can be used to train models that increase in quality as more unlabeled data become available, making efficient use of clinician resources.

Our results also suggest specific situations in which modeling the labeling process can improve the performance of weakly supervised models in medical imaging and monitoring. In the CXR and EXR applications on which generative and discriminative modeling over the text was able to provide more useful sets of labels for target modality model training, clinician LFs tended to be quite accurate but relatively low-coverage. Such a pattern would be expected in screening applications such as radiograph triage wherein the variety of pathologies that can appear on a scan is large.[7,32] Hemorrhage detection on HCT and seizure detection on EEG, on the other hand, are more targeted detection applications wherein clinicians are tasked with identifying a specific pathology. In these cases, clinicians tend to refer to the pathology in question in their reports in very consistent ways, which makes the LFs they provide both accurate and high coverage. Our results suggest that simple majority vote over report text LFs may often be sufficient for building highly accurate detection models for specialized diagnostic protocols, while additional generative or discriminative modeling could provide substantial benefit for training machine-learning models on target modalities intended for general screening.

Several limitations should be considered when interpreting the results of this study. First, because we use data sourced from a single hospital, our results do not address cross-institutional model validation. While substantial resources are required to de-identify and make publicly available datasets of the sizes considered here, we hope that continued progress on this front will allow for future work that addresses this important question. Second, although the statistical estimation techniques underlying cross-modal data programming do handle the multi-class setting, this work focuses on well-defined binary tasks. Third, cross-modal data programming is limited to cases wherein an auxiliary modality exists, is readily available, and is amenable to rapid LF specification. Finally, our study assumes the existence of reference neural network architectures for each

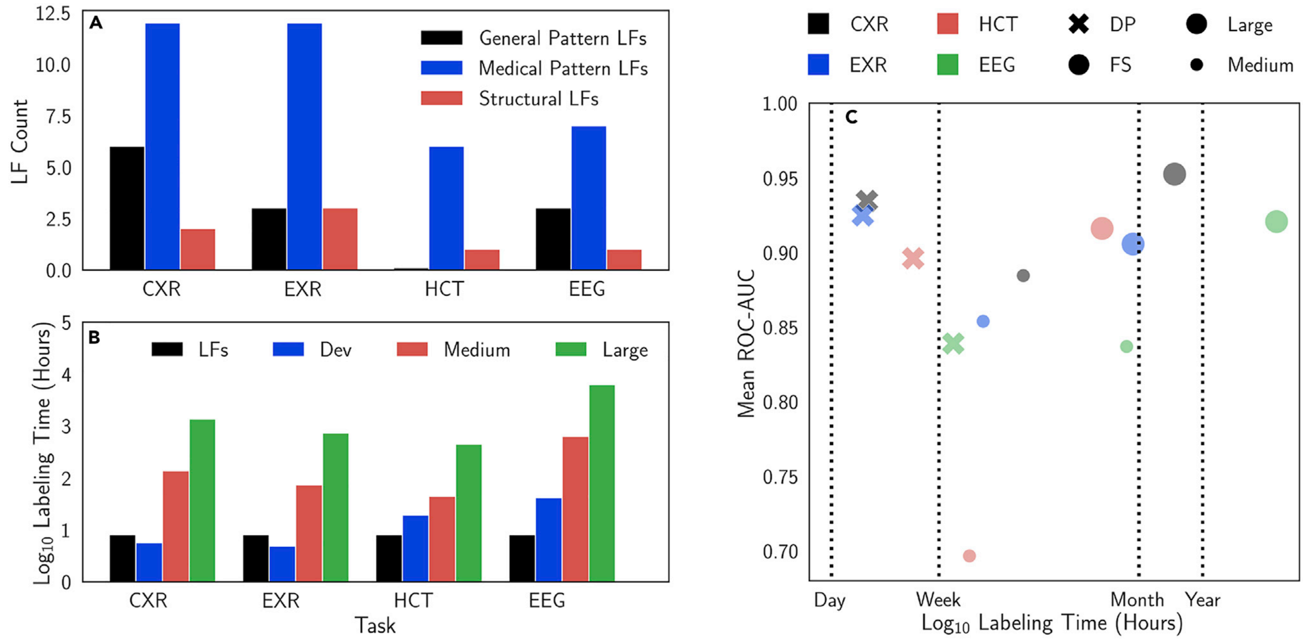

Figure 5. Analysis of Labeling Function Types and Time-versus-Performance Tradeoffs

(A and B) Labeling function (LF) types (A); labeling time for datasets describing chest (CXR) and extremity (EXR) radiographs, head CT (HCT), and electroencephalography (EEG) (B). Labeling times are presented for the small development set (Dev) of several hundred examples, the Large fully supervised dataset (i.e., physician-years of labeling time), and the Medium fully supervised dataset (i.e., physician-months of labeling time). See Table 1 for additional details on dataset sizes. Hand-labeling times were estimated using median read times of 1 min 38 s per CXR, 1 min 26 s per EXR, 6 min 38 s per HCT, and 12 min 30 s per EEG drawn from reported values in the literature.[39,40] These estimates are conservative because they assume that only a single clinician contributed to reading each case.
(C) Labeling time versus performance in the context of dataset size, the task, and the type of supervision. Cross-modal data programming (DP) often yields models similar in performance to those trained on Large hand-labeled datasets (FS) but using a fraction of the labeling time.

diagnostic modality; while this is often valid in computer vision, additional resources may be required to create high-performance models for other diagnostic modalities. Addressing each of these points would be a natural and valuable direction for future research.

Remaining challenges associated with translating this work to clinical practice mirror those usually faced by machine learning systems for decision support in medicine. In addition to well-documented regulatory hurdles, these include integrating rapid model iteration and data collection workflows into hospitals and care centers, designing robust testing infrastructures to support continual model improvement while guarding against critical failure modes, and training physicians to best make use of these algorithms.[43] Indeed, our specific aim with this work is to reduce these barriers. By demonstrating that models trained using cross-modal weak supervision can perform comparably with their fully supervised counterparts on four clinically relevant tasks with only days of development time, our results suggest that high-performance machine learning models can be developed quickly enough to support more rapid clinical deployment. This ability to shorten the model development cycle has other practical benefits, as models could be rapidly retrained using cross-modal weak supervision to address important issues such as failures on critical subsets, changes in patient population demography, or updates to scanning hardware. It is our hope that the conclusions of this work will provide empirical support for deploying higher-level, more practical methods of generating training data for modern machine learning algorithms in the clinical setting, translating advances that have had substantial impact on the technology industry to improve patient care.

EXPERIMENTAL PROCEDURES

Study Design

The purpose of this study is to assess the hypothesis that cross-modal weak supervision techniques such as cross-modal data programming can enable rapid construction of useful machine learning models across diverse clinical-use cases. Each dataset analyzed here was retrospectively collected from our institution, and Institutional Review Board (IRB) approval was obtained from our institution (Stanford University) in each case. In each dataset, different patients were randomly assigned to training, development, and held-out test sets, and subsets of the training set were chosen via random sampling. The study was blinded in the sense that the study authors were unaware of which patients were in the train, development, and test sets.

Text models were trained using the Snorkel[30] software package and standard tools in PyTorch. For each application, a 20-trial random hyperparameter search was performed to determine final values for learning rate and ℓ_2 regularization to use for training the GM described in Equation 1. Five separate GMs were then trained using procedures documented in the Snorkel codebase.[30] The output of each GM training procedure was then used to train a discriminative bidirectional LSTM with attention[44] wherein the input was the raw text and the output was the generative model label. Hyperparameters for the LSTM for each application were determined via 20-trial random search; this same set of hyperparameters was used to train all LSTMs within each application. Standard tools in PyTorch were used for tokenization, word embeddings were initialized randomly, and each model consisted of a single bidirectional LSTM layer with hidden size 128. LSTM models were trained using

CellPress
OPEN ACCESS

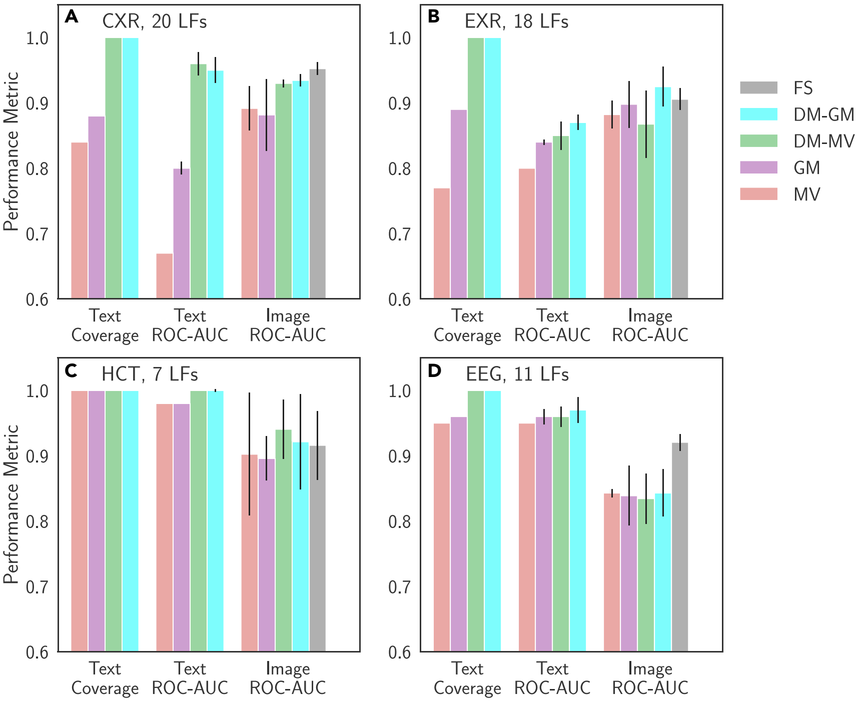

Figure 6. Model Performance versus Supervision Type on Auxiliary (Text) and Target (Image or Signal) Domains

We analyzed the performance of majority vote (MV) of the labeling functions (LFs), a generative model trained on these LFs (GM), an LSTM trained to map the raw text to the MV output (DM-MV), an LSTM trained to map the raw text to the GM output (DM-GM), and hand-labeled full supervision (FS). Text model performance is evaluated on the development set, as coverage and ROC-AUC on this set are used in the cross-modal data programming heuristic optimizer. Target modality performance is evaluated on the held-out test set. We present results for CXR (A), EXR (B), HCT (C), and EEG (D). Note that MV text results and coverage results are deterministic. Error bars are 95% confidence intervals from five runs with different random seeds.

the Adam optimizer until validation loss had not decreased for more than three epochs.

Details of dataset composition, preprocessing routines, training procedures, and neural network architectures for each target modality model are provided below. LFs for each application and an annotated, functional Jupyter notebook tutorial applying the cross-modal data-programming technique to a publicly available CXR dataset are provided in Supplemental Experimental Procedures.

Automated Triage of Frontal Chest Radiographs

As the demand for imaging services increases, automated triage for common diagnostics such as CXRs is expected to become an increasingly important part of radiological workflows.[5,7] Our frontal chest radiography dataset comprises 50,000 examples wherein each instance contains an image, a free-text radiology report, and a prospective normal or abnormal label provided by a single radiologist at the time of interpretation. Each data point describes a unique patient, and the dataset balance is 80% abnormal. The fully supervised (hand-labeled) models are trained using these prospective labels. For both fully and weakly supervised models, we use a development set size of 200 prospectively labeled images for cross-validation during the training process and evaluate on the same 533-image held-out test dataset as Dunnmon et al.,[7] which is labeled by blinded consensus of two radiologists with 5 and 20 years of training. Examples of CXRs can be found in Figure 2A.

For this task, a radiology fellow wrote 20 LFs over the text in less than 8 h of cumulative clinician time, using a labeled development set of 200 reports. These LFs were implemented in Python with several hours of assistance from a computer science graduate student. These LFs represent a combination of pattern-matching, comparison with known knowledge bases, and domain-specific cues. In the context of chest radiography, the impression and findings sections of the radiology report tend to be the most informative; thus, the majority of LFs considered text within these sections. Once LFs had been generated, tools within the Snorkel software package were used to generate sets of probabilistic training labels.[30] Note that the particular set of LFs used for this application is not meant to be prescriptive or exhaustive; rather, it represents the output of a real-world effort to create useful rules for programmatic labeling of data with domain experts in a reasonable period of time.

The image model used for this task was an 18-layer Residual Network (ResNet-18) with a sigmoid nonlinearity on top of a single-neuron final layer, which yields near-state-of-the-art results on this dataset at a modest computational cost.[7,9] The model was implemented using the PyTorch software framework and was initialized using weights pretrained on the ImageNet database.[11,45] Models were trained on a single Tesla P100 GPU using the Adam optimizer with default parameters and early stopping, an initial learning rate of 0.001, batch size of 72, learning rate decay rate of 0.1 on plateau in the validation loss, weight decay (i.e., ℓ_2 regularization) value of 0.005, and the binary cross-entropy loss function. We train models on the full dataset for 20 epochs with early stopping and use an equivalent number of batches for models trained on subsets of the dataset. Images were preprocessed using histogram equalization, downsampled to 224 × 224 resolution, Z-score normalized using global mean and standard deviation values computed across the dataset, and replicated over three channels (for compatibility with a model originally for RGB images) before injection into the training loop. Each model over 50,000 images took approximately 6 h to train.

Because each image is associated with a unique report, evaluation is performed on a simple image-by-image basis, using ROC-AUC as the evaluation metric.

Automated Triage of Extremity Radiograph Series

Musculoskeletal disorders are generally diagnosed from imaging, and their substantial prevalence makes them a natural target for automated triage systems; in this work, we specifically focus on detecting musculoskeletal disorders of the knee from multiple-view radiograph series. We originally obtained a dataset of 3,564 patient exams prospectively labeled as normal or abnormal in the same manner as described for chest radiography, containing a total of 37,633 individual images and 3,564 reports. A single exam can have a variable number of radiographs across different views, as collection protocols are not standard for this type of exam. The dataset was pulled from the PACS system in such a way that it contains a 50:50 distribution of normal and abnormal cases (as indicated by the prospective label). We split the dataset such that images from a randomly sampled 3,008-patient cohort are available for training both fully supervised and weakly supervised models, with images from 200 patients available for cross-validation. We then obtained retrospective labels for each image in a held-out test set of 356 patient exams (3,718 images) where a single radiologist with 9 years of training provided a normal or abnormal label with no time constraints. We used this set of 356 exams for evaluation on a patient-by-patient basis. Examples of knee radiographs can be found in Figure 2B.

For this task, a single radiology fellow wrote the 18 LFs within 8 h of cumulative clinician time, which were implemented in Python with several hours of assistance from a computer science graduate student using a development set of 200 labeled reports. These LFs use a variety of pattern-matching and

semantic cues to identify reports as either normal or abnormal. While there is some overlap between these LFs and those for the CXRs (e.g., the length of the report), LFs nonetheless had to be adapted for this specific application. Probabilistic labels were then generated from these LFs using tools in the Snorkel software package.[30] Note that this application represents an MIL setting wherein the label extracted from a report is applied to all images in the study, but it is unknown which of these images actually contains the abnormality.

The image model for this task takes a single radiograph as input and outputs the probability that this radiograph is abnormal. We use a 50-layer Residual Network (ResNet-50) architecture modified to emit a binary classification result on each radiograph in the training set, and leverage ImageNet-pretrained weights for model initialization.[9,45] The model is implemented using the Keras software package[46] and trained using the Adam optimizer with early stopping, a learning rate of $\alpha = 0.0001$ with learning rate decay rate of 0.1 on plateau in the validation loss, a weight decay value of 0.005, and the binary cross-entropy loss function. We train models on the full dataset for 30 epochs with early stopping and use an equivalent number of batches for models trained on subsets of the dataset. Images were preprocessed using histogram equalization, downsampled to 224 × 224 resolution, Z-score normalized using global mean and standard deviation values computed across the dataset, and replicated over three channels (for compatibility with a model originally for RGB images) before injection into the training loop. Batch size was set at 60 radiographs, the maximum possible on the single 1080 Ti GPU that was used to train each model. Models over 30,000 training images took approximately 6 h to train. For each exam in the test set, we compute an output score by taking the mean of the image model outputs for all radiographs in the exam following Rajpurkar et al.[32]

Intracranial Hemorrhage Detection on Computed Tomography

The problem of rapid intracranial hemorrhage detection on CT of the head (HCT) represents an important task in clinical radiology to expedite clinical triage and care.[47] To create a dataset describing a binary task of intracranial hemorrhage detection on HCT, we acquired 5,582 non-contrast HCT studies performed between the years 2001 and 2014 from our institution's PACS system. Each study was evaluated for a series containing 5-mm axial CT slices with greater than 29 slices and fewer than 45 slices. For each study containing the requisite 5-mm axial series, the series was padded with additional homogeneous images with Hounsfield units of 0 such that each series contained 44 CT slices. The center 32 slices were then selected for automated analysis. The final dataset contains 4,340 studies preprocessed in this way, and 340 of these examples are provided with a ground truth label at the scan level (cf. the slice level[6]) confirmed by consensus of two radiology fellows. Of these hand-labeled scans, 50% (170 scans) are allocated for cross-validation during end-model training while the rest are used for evaluation. Scan-level hand labels for the 4,000 images that support assessment of model performance using fully supervised training were provided via single-annotator reads of each report. An example set of 32 CT slices for both hemorrhage and non-hemorrhage cases can be found in Figure 2C.

The seven LFs for the hemorrhage detection task were written in Python entirely by a single radiology resident in less than 8 h of cumulative development time using a labeled development set of 200 reports. Notably, these LFs programmatically combine many possible expressions for normality or hemorrhage that are drawn from this radiologist's personal experience. Again, probabilistic labels are generated from these LFs using tools in the Snorkel software package.[30]

The hemorrhage detection task fits naturally with the framework of MIL because we have a single label per CT scan, each of which contains 32 image slices. Furthermore, if a CT image is labeled as positive, there must exist at least one slice that contains evidence of a hemorrhage. We leverage the attention-based MIL approach recommended by Ilse et al.[35] for this task. Specifically, we embed each instance (i.e., each slice) into a feature space and subsequently learn a weighted average off all instance (i.e., slice) embeddings corresponding to a single bag of instances (i.e., a single CT scan). These weights are learned using a two-layer neural network known as an "attention layer." Classification is then performed using the attention-weighted combination of all instances (i.e., all CT slices).

Our attention-based MIL model uses a randomly initialized ResNet-18 encoder with an output size of 50. Each slice is downsampled to dimensions of 224 × 224 and Z-score normalized using global mean and standard deviation values computed across the dataset. Model training is accomplished using the stochastic gradient descent optimizer in PyTorch with a learning rate that was initially set to a high value of 0.1 and reduced upon plateaus in the validation loss, a momentum of 0.9, and a weight decay value of 0.005. Models over the full dataset were trained for 30 epochs with early stopping, and use an equivalent number of batches for models trained on subsets of the dataset. Batch size was set at 12 CT scans, the maximum possible using the single Tesla P100 GPU that was used to train each model. Training each model on the full set of images took approximately 6 h.

Because only 39 of the 340 consensus-labeled examples were positive for hemorrhage, the end-model performance metrics could be sensitive to the random splitting of the test and development datasets. We therefore carry out a cross-validation procedure where we repeat the stratified 50:50 development set/test set split using the 340 gold-labeled data points, and analyze average model performance over five trials with different random seeds, where one of the seeded operations was the development set/test set split.

Seizure Monitoring on Electroencephalography

One of the most common tasks performed using EEG is epileptic seizure detection, where an epileptologist examines large amounts of time-series data to determine whether the repeated, uncontrolled electrical discharges suggestive of seizure activity have occurred. Our EEG dataset comprises 36,644 pediatric EEG signals from our institution along with 9,496 EEG reports. Each EEG report can reference multiple signals, and each signal can be referenced by multiple EEG reports. Each signal is annotated by an EEG technician with onset times of possible seizures, which we treat as full hand-labeled supervision for a machine-learning model for seizure detection. To ensure consistency across exams, each of which could have a unique sensor alignment, we use only signals from the 19 electrodes in the standard 10-20 International EEG configuration, which forms a subset of the electrodes deployed to every patient at our institution. Voltage readings from each channel are sampled at 200 Hz. We approach the seizure-onset detection problem as a clip-level classification problem over 12-s clips of the full time series. Our model maps an input $x \in R^{2400x19}$ to a single output indicating the probability of seizure onset in that clip. An example set of EEG signals can be found in Figure 2D.

The 11 LFs used to determine whether a given EEG clip contains seizure onset were written collaboratively by a clinical neurologist and a postdoctoral computer scientist over the course of less than 8 cumulative hours of dedicated time using a labeled development set of 200 reports. These LFs simultaneously leverage the structure of the EEG report along with unstructured information contained in the raw text, which can often cover several paragraphs. The Snorkel software package is then used to create probabilistic labels for each report.[30] Due to the length of these reports and their highly variable structure, the LFs for this application represent a particularly compelling example of how domain-specific knowledge can be used to inform heuristic development.

Because these reports refer to entire signals rather than to specific clips, a small hand-labeled EEG clip dataset for cross-validation and end-model evaluation was created by a pediatric clinical neurologist with 10 years of experience. This dataset contains 350 12-s clips representative of seizure onset; 100 of these positive examples are allocated to a development set for cross-validating the end model and 250 are allocated to the test set for final evaluation. Sets used for both cross-validation and evaluation were made to contain an 80% fraction of clips without seizure onset by randomly sampling clips from signals in each set confirmed to contain no evidence of seizure. We use a densely connected Inception architecture inspired by Roy et al.[36] for seizure-onset detection. This modeling approach combines the most compelling aspects of the InceptionNet[48] and DenseNet[37] architectures, namely the extraction of convolutional features at multiple granularities at each layer combined with concatenation of each filter bank with all of those preceding it. To address the issue of extreme class imbalance caused by the low frequency of clips containing seizure onset even in EEG signals that contain a seizure, we use a simple filtering process analogous to common techniques used in the information extraction literature for candidate extraction for weakly supervised models.[30] Specifically, we construct a candidate extractor that is a three-layer neural network operating on a set of 551 features reported to be useful for seizure detection from the literature.[49] This candidate extractor, which is trained on the development set, is executed over all signals with an

CellPress
OPEN ACCESS

associated report that is weakly labeled as containing a seizure to provide clips that are positive for seizure onset with high probability, while negative clips are randomly sampled from signals with no associated positive report. The candidate extractor cutoff was tuned using the development set such that precision was 100% and recall was 15%; because we have such a large unlabeled dataset, we are able to optimize for this high level of precision while still obtaining a large set of positive examples.

Model training is accomplished using the Adam optimizer in PyTorch, learning rate was initially set to a value of 1×10^{-6} and reduced by a factor of 0.1 upon plateaus in the validation loss, and a dropout probability of 0.2 was applied to the last layer. We train models on the full dataset for 30 epochs with early stopping and use an equivalent number of batches for models trained on subsets of the dataset. Plentiful negative examples were undersampled such that the train set contained 50% positive examples. Batch size was set at ten EEG signals, the maximum possible using the single Tesla P100 GPU that was used to train each model. Training each model on the full set of signals took approximately 12 h.

Statistical Analysis

All statistical tests performed in this work are two-tailed DeLong non-parametric tests[41] to evaluate the equivalence of ROC-AUC values. These tests were implemented using the R package pROC accessed via the Python package rpy2. A statistical significance (α) threshold of 0.05 was used for all reported tests. All ROC-AUC values were computed using the entire test sets described above. Confidence intervals reported in plots are standard 95% confidence intervals assuming a normally distributed population.

DATA AND CODE AVAILABILITY

Raw data for Figures 3, 4, 5, and 6 are available from the authors. Protected Health Information restrictions apply to the availability of the raw clinical datasets presented here, which were used under IRB approval for use only in the current study. As a result, these datasets are not yet publicly available. We have, however, made available the LFs used for each application as well as a full working demonstration of the cross-modal weak supervision pipeline using a small public dataset containing CXRs, ground truth labels, and text reports in Supplemental Experimental Procedures. The code in this demonstration exactly mirrors that used for analyses presented in this paper.

All code used for this study uses open-source Python packages. We have made available the LFs themselves as well as a full annotated demonstration of the cross-modal weak supervision pipeline using a small public dataset containing CXRs and text reports in an online repository as described in Supplemental Experimental Procedures. Technical background and usage information on the Snorkel software package used heavily in this work can be found at snorkel.stanford.edu.

SUPPLEMENTAL INFORMATION

Supplemental Information can be found online at https://doi.org/10.1016/j.patter.2020.100019.

ACKNOWLEDGMENTS

We would like to extend deep thanks to Jason Fries, James Priest, Avner May, Charles Kuang, Fred Sala, Sarah Hooper, and Nimit Sohoni for helpful discussions as we formulated the manuscript. We gratefully acknowledge the support of DARPA under nos. FA87501720095 (D3M) and FA86501827865 (SDH); NIH under nos. U54EB020405 (Mobilize), 1U01CA190214, and 1U01CA187947; NSF under nos. CCF1763315 (Beyond Sparsity) and CCF1563078 (Volume to Velocity); ONR under no. N000141712266 (Unifying Weak Supervision); the National Library of Medicine under no. R01LM01296601; the Stanford Child Health Research Institute under no. UL1-TR001085; the Stanford Human-Centered Artificial Intelligence Seed Grant; the Moore Foundation; NXP; Xilinx; LETI-CEA; Intel; Google; Microsoft; NEC; Toshiba; TSMC; ARM; Hitachi; BASF; Accenture; Ericsson; Qualcomm; Analog Devices; the Okawa Foundation; American Family Insurance; LVIS; and members of the Stanford DAWN project: Teradata, Facebook, Google, Ant Financial, NEC, VMWare, and InfoSys. J.A.D. was supported by the Intelligence Community Postdoctoral Fellowship, A.J.R. and K.S. were supported by the Stanford Interdisciplinary Graduate Fellowship, and H.S. was supported by an RSNA Research Fellow grant from the Radiological Society of North America and NIH no. T32-CA009695 (Stanford Cancer Imaging Training Program). This research used data or services provided by STARR, "STAnford medicine Research data Repository," a clinical data warehouse containing live Epic Clarity warehouse data from Stanford Health Care, the Stanford Children's Hospital, the University Healthcare Alliance, and Packard Children's Health Alliance clinics and other auxiliary data from hospital applications such as radiology PACS. STARR is made possible by Stanford School of Medicine Research Office. The US Government is authorized to reproduce and distribute reprints for Governmental purposes notwithstanding any copyright notation thereon. Any opinions, findings, and conclusions or recommendations expressed in this material are those of the authors and do not necessarily reflect the views, policies, or endorsements, either expressed or implied, of DARPA, NIH, ONR, or the US Government.

AUTHOR CONTRIBUTIONS

A.J.R., J.A.D., D.L.R., and C.R. conceptualized the study. J.A.D., A.J.R., R.G., N.K., K.S., D.L.R., and C.R. contributed to experimental design while J.A.D., A.J.R., N.K., and K.S. wrote computer code and performed experiments. M.P.L., R.G., C.L.-M., M.M., H.S., and D.L.R. provided labeled data and clinical expertise for the study. R.G., N.K., C.L.-M., and J.A.D. contributed to LF development and tuning. J.A.D. and A.J.R. prepared the manuscript. All authors contributed to manuscript review.

DECLARATION OF INTERESTS

J.A.D. is an advisor of Snorkel AI. A.J.R. is a founder and shareholder of Snorkel AI. N.K. is a shareholder of Bunkerhill Health. M.M. has received consulting fees from Ceribell. M.P.L. is a shareholder and advisor of Nines, SegMed, and Bunkerhill Health. C.R. is a founder and shareholder of Snorkel AI.

Received: November 12, 2019
Revised: February 9, 2020
Accepted: March 17, 2020
Published: April 28, 2020

REFERENCES

1. Bychkov, D., Linder, N., Turkki, R., Nordling, S., Kovanen, P.E., Verrill, C., Walliander, M., Lundin, M., Haglund, C., and Lundin, J. (2018). Deep learning based tissue analysis predicts outcome in colorectal cancer. Sci. Rep. *8*, 3395.
2. Yu, K.-H., Zhang, C., Berry, G.J., Altman, R.B., Ré, C., Rubin, D.L., and Snyder, M. (2016). Predicting non-small cell lung cancer prognosis by fully automated microscopic pathology image features. Nat. Commun. *7*, 12474.
3. Esteva, A., Kuprel, B., Novoa, R.A., Ko, J., Swetter, S.M., Blau, H.M., and Thrun, S. (2017). Dermatologist-level classification of skin cancer with deep neural networks. Nature *542*, 115–118.
4. Gulshan, V., Peng, L., Coram, M., Stumpe, M.C., Wu, D., Narayanaswamy, A., Venugopalan, S., Widner, K., Madams, T., Cuadros, J., et al. (2016). Development and validation of a deep learning algorithm for detection of diabetic retinopathy in retinal fundus photographs. JAMA *316*, 2402.
5. Titano, J.J., Badgeley, M., Schefflein, J., Pain, M., Su, A., Cai, M., Swinburne, N., Zech, J., Kim, J., Bederson, J., et al. (2018). Automated deep-neural-network surveillance of cranial images for acute neurologic events. Nat. Med. *24*, 1337–1341.
6. Lee, H., Yune, S., Mansouri, M., Kim, M., Tajmir, S.H., Guerrier, C.E., Ebert, S.A., Pomerantz, S.R., Romero, J.M., Kamalian, S., et al. (2019). An explainable deep-learning algorithm for the detection of acute intracranial haemorrhage from small datasets. Nat. Biomed. Eng. *3*, 173–182.

7. Dunnmon, J.A., Yi, D., Langlotz, C.P., Ré, C., Rubin, D.L., and Lungren, M.P. (2019). Assessment of convolutional neural networks for automated classification of chest radiographs. Radiology *290*, 537–544.

8. Rajpurkar, P., Irvin, J., Ball, R.L., Zhu, K., Yang, B., Mehta, H., Duan, T., Ding, D., Bagul, A., Langlotz, C.P., et al. (2018). Deep learning for chest radiograph diagnosis: a retrospective comparison of the CheXNeXt algorithm to practicing radiologists. PLoS Med. *15*, e1002686.

9. He, K., Zhang, X., Ren, S., and Sun, J. (2016). Deep residual learning for image recognition. 2016 IEEE Conference on Computer Vision and Pattern Recognition (CVPR), 770–778.

10. Abadi, M., Barham, P., Chen, J., Chen, Z., Davis, A., Dean, J., Devin, M., Ghemawat, S., Irving, G., Isard, M., et al. (2016). Tensorflow: a system for large-scale machine learning. Symp. Oper. Syst. Des. Implement. *16*, 265–283.

11. Paszke, A., Gross, S., Chintala, S., Chanan, G., Yang, E., Facebook, Z.D., Research, A.I., Lin, Z., Desmaison, A., Antiga, L., et al. (2019). Automatic differentiation in PyTorch. Adv. Neural Inf. Process. Syst. *32*, 8024–8035.

12. Esteva, A., Robicquet, A., Ramsundar, B., Kuleshov, V., DePristo, M., Chou, K., Cui, C., Corrado, G., Thrun, S., and Dean, J. (2019). A guide to deep learning in healthcare. Nat. Med. *25*, 24–29.

13. Etzioni, O., Cafarella, M., Downey, D., Kok, S., Popescu, A.-M., Shaked, T., Soderland, S., Weld, D.S., and Yates, A. (2004). Web-scale information extraction in KnowItAll. WWW '04: Proceedings of the 13th International Conference on World Wide Web, 100–110.

14. Hearst, M.A. (1992). Automatic acquisition of hyponyms from large text corpora. Proceedings of the 14th Conference on Computational Linguistics, 539–545.

15. Blum, A., and Mitchell, T. (1998). Combining labeled and unlabeled data with co-training. Proceedings of the 11th Annual Conference on Computational Learning Theory, 92–100.

16. Bach, S.H., Rodriguez, D., Liu, Y., Luo, C., Shao, H., Xia, C., Sen, S., Ratner, A., Hancock, B., Alborzi, H., et al. (2019). Snorkel DryBell: a case study in deploying weak supervision at industrial scale. Proceedings of the 2019 International Conference on Management of Data, 362–375.

17. Dehghani, M., Severyn, A., Rothe, S., and Kamps, J. (2017). Learning to learn from weak supervision by full supervision, NIPS Workshop on Meta-Learning (MetaLearn 2017) http://metalearning.ml/2017/papers/metalearn17_dehghani.pdf.

18. Liang, C., Berant, J., Le, Q., Forbus, K.D., and Lao, N. (2017). Neural symbolic machines: learning semantic parsers on freebase with weak supervision. Proceedings of the 55th Annual Meeting of the Association for Computational Linguistics, 23–33.

19. Dehghani, M., Zamani, H., Severyn, A., Kamps, J., and Croft, W.B. (2017). Neural ranking models with weak supervision. Proceedings of the 40th International ACM SIGIR, 65–74.

20. Mahajan, D., Girshick, R., Ramanathan, V., He, K., Paluri, M., Li, Y., Bharambe, A., and van der Maaten, L. (2018). Exploring the limits of weakly supervised pretraining. In Computer Vision—ECCV 2018, V. Ferrari, M. Hebert, C. Sminchisescu, and Y. Weiss, eds. (Springer), pp. 185–201.

21. Jia, Z., Huang, X., Eric, I., Chang, C., and Xu, Y. (2017). Constrained deep weak supervision for histopathology image segmentation. IEEE Trans. Med. Imaging *36*, 2376–2388.

22. Sun, C., Shrivastava, A., Singh, S., and Gupta, A. (2017). Revisiting Unreasonable effectiveness of data in deep learning era. In Proceedings of the IEEE International Conference on Computer Vision, pp. 843–852.

23. Wang, X., Peng, Y., Lu, L., Lu, Z., Bagheri, M., and Summers, R.M. (2017). Chest X-ray8: hospital-scale chest X-ray database and benchmarks on weakly supervised classification and localization of common thorax diseases. In Proceedings of the 2017 IEEE Conference on Computer Vision and Pattern Recognition (CVPR) (IEEE), pp. 3462–3471.

24. Peng, Y., Wang, X., Lu, L., Bagheri, M., Summers, R., and Lu, Z. (2018). NegBio: a high-performance tool for negation and uncertainty detection in radiology reports. Proceedings of the American Medical Informatics Association Summits on Translational Science 2017, 188.

25. Irvin, J., Rajpurkar, P., Ko, M., Yu, Y., Ciurea-Ilcus, S., Chute, C., Marklund, H., Haghgoo, B., Ball, R., Shpanskaya, K., et al. (2019). CheXpert: a large chest radiograph dataset with uncertainty labels and expert comparison. In Proceedings of AAAI Conference on Artificial Intelligence, *33* (AAAI Press), pp. 590–597.

26. Zech, J., Pain, M., Titano, J., Badgeley, M., Schefflein, J., Su, A., Costa, A., Bederson, J., Lehar, J., and Oermann, E.K. (2018). Natural language-based machine learning models for the annotation of clinical radiology reports. Radiology *287*, 570–580.

27. Leaman, R., Islamaj Dougan, R., and Lu, Z. (2013). DNorm: disease name normalization with pairwise learning to rank. Bioinformatics *29*, 2909–2917.

28. Aronson, A.R. (2001). Effective mapping of biomedical text to the UMLS Metathesaurus: the MetaMap program. In Proceedings of AMIA Symposium, S. Bakken, ed. (Hanley & Belfus), pp. 17–21.

29. Ratner, A.J., Sa, C.M., De Wu, S., Selsam, D., and Ré, C. (2016). Data programming: creating large training sets, quickly. Adv. Neural Inf. Process. Syst. *29*, 3567–3575.

30. Ratner, A., Bach, S.H., Ehrenberg, H., Fries, J., Wu, S., and Ré, C. (2020). Snorkel: rapid training data creation with weak supervision. VLDB J. *29*, 709–730.

31. Ratner, A., Hancock, B., Dunnmon, J., Sala, F., Pandey, S., and Ré, C. (2019). Training complex models with multi-task weak supervision. In Proceedings of AAAI Conference on Artificial Intelligence, *33* (AAAI Press), pp. 4763–4771.

32. Rajpurkar, P., Irvin, J., Bagul, A., Ding, D., Duan, T., Mehta, H., Yang, B., Zhu, K., Laird, D., Ball, R.L., et al. (2018). MURA: large dataset for abnormality detection in musculoskeletal radiographs. In 1st Conference on Medical Imaging with Deep Learning. https://arxiv.org/abs/1712.06957.

33. Obeid, I., and Picone, J. (2016). The Temple University Hospital EEG data corpus. Front. Neurosci. *10*, 196.

34. Hochreiter, S., and Schmidhuber, J. (1997). Long short-term memory. Neural Comput. *9*, 1735–1780.

35. Ilse, M., Tomczak, J.M., and Welling, M. (2018). Attention-based deep multiple instance learning. In International Conference on Machine Learning, *80*, J. Dy and A. Krause, eds. *(PMLR), pp. 2127–2136*.

36. Roy, S., Kiral-Kornek, I., and Harrer, S. (2019). ChronoNet: a deep recurrent neural network for abnormal EEG identification. Proceedings of Conference on Artificial Intelligence in Medicine, Europe, 47–56.

37. Huang, G., Liu, Z., Van Der Maaten, L., and Weinberger, K.Q. (2017). Densely connected convolutional networks. IEEE Conference on Computer Vision and Pattern Recognition (CVPR), 2261–2269.

38. National Institutes of Health (2014). Open-i: open access biomedical image search engine. Choice Rev. Online *51*, 51–5633, 51–5633.

39. Cowan, I.A., MacDonald, S.L., and Floyd, R.A. (2013). Measuring and managing radiologist workload: measuring radiologist reporting times using data from a Radiology Information System. J. Med. Imaging Radiat. Oncol. *57*, 558–566.

40. Brogger, J., Eichele, T., Aanestad, E., Olberg, H., Hjelland, I., and Aurlien, H. (2018). Visual EEG reviewing times with SCORE EEG. Clin. Neurophysiol. Pract. *3*, 59–64.

41. Delong, E.R., Delong, D.M., and Clarke, D.L. (1988). Comparing the areas under two or more correlated receiver operating characteristic curves: a nonparametric approach. Biometrics *44*, 837–845.

42. Acharya, U.R., Oh, S.L., Hagiwara, Y., Tan, J.H., and Adeli, H. (2018). Deep convolutional neural network for the automated detection and diagnosis of seizure using EEG signals. Comput. Biol. Med. *100*, 270–278.

43. He, J., Baxter, S.L., Xu, J., Xu, J., Zhou, X., and Zhang, K. (2019). The practical implementation of artificial intelligence technologies in medicine. Nat. Med. *25*, 30–36.

44. Zhou, P., Shi, W., Tian, J., Qi, Z., Li, B., Hao, H., and Xu, B. (2016). Attention-based bidirectional long short-term memory networks for relation classification. In 54th Annual Meeting of the Association for

CellPress
OPEN ACCESS

Computational Linguistics, ACL 2016—Short Papers (Association for Computational Linguistics), pp. 207–212.

45. Jia Deng, Wei Dong, Socher, R., Li-Jia Li, Kai Li, and Li Fei-Fei (2009). ImageNet: a large-scale hierarchical image database. IEEE Conference on Computer Vision and Pattern Recognition (CVPR), 248–255.

46. Chollet, F. (2015). Keras. https://github.com/fchollet/keras.

47. Saver, J.L. (2006). Time is brain-quantified. Stroke *37*, 263–266.

48. Szegedy, C., Liu, W., Jia, Y., Sermanet, P., Reed, S., Anguelov, D., et al. (2015). Going deeper with convolutions. IEEE Conference on Computer Vision and Pattern Recognition (CVPR), 1–9.

49. Boubchir, L., Daachi, B., and Pangracious, V. (2017). A review of feature extraction for EEG epileptic seizure detection and classification. 40th International Conference on Telecommunications and Signal Processing, 456–460.

PATTER, Volume *1*

Supplemental Information

Cross-Modal Data Programming

Enables Rapid Medical Machine Learning

Jared A. Dunnmon, Alexander J. Ratner, Khaled Saab, Nishith Khandwala, Matthew Markert, Hersh Sagreiya, Roger Goldman, Christopher Lee-Messer, Matthew P. Lungren, Daniel L. Rubin, and Christopher Ré

Supplemental Information

Supplemental Data Items

Dataset	CXR	EXR	HCT	EEG
Large FS vs. Large DP	0.3821	0.012	0.6223	0.0002
Medium FS vs. Large DP	0.0004	0.0002	0.0006	0.2670

Table S1. Statistical analysis of cross-modal data programming versus full supervision. P-values from the two-tailed DeLong non-parametric test[36] comparing ROC curves from median models. These results demonstrate that median CXR and HCT models supervised with cross-modal data programming (DP) are not statistically distinguishable from those supervised with the Large fully supervised (i.e. hand-labeled) set ($p > 0.35$), and are significantly different (DeLong $p < 0.001$) than those supervised with the Medium fully supervised set (see Table 1 for dataset size definitions). For EXR, median models supervised with cross-modal data programming were statistically superior to those trained using the Large fully supervised set (DeLong $p < 0.05$). For EEG, median models supervised with cross-modal data programming were statistically different than those supervised using the Large supervised set (DeLong $p < 0.05$), but not distinguishable from those trained using the Medium fully supervised set (DeLong $p > 0.25$).

Dataset	CXR	EXR	HCT	EEG
Metric	ROC-AUC	ROC-AUC	ROC-AUC	Weighted F1
Literature	0.95[7]	0.93[32]	0.96[6]	0.88[50]
Ours	0.95	0.91	0.92	0.85

Table S2. Comparison of fully supervised models to studies in the literature. Performance of fully supervised models presented in this work compared to best reported values from similar studies in the literature. For CXR, our results are equivalent to those of Dunnmon et al.,[7] for EXR our results are within 2 points ROC-AUC of Rajpurkar et al.,[32] for HCT our results are within 4 points ROC-AUC of Lee et al.,[6] and our results are within 3 points weighted F1 of Asif et al.[50] for EEG. Comparisons for CXR and EXR are direct, as similar datasets and labeling schema were used. Comparisons to HCT are conservative, as our model uses scan-level supervision, while Lee et al.[6] uses far more detailed slice-level supervision; the most relevant work using scan-level supervision for ICH detection is that of Jnawali et al.,[51] which attains an ROC-AUC of 0.86. In EEG, most studies report results using small numbers of patients;[37] modern studies using the Temple University Hospital EEG corpus are most relevant to our work,[33,50,52] but use costly retrospective multi-class supervision rather than binary technician labels, which are commonly obtained in the course of clinical practice.

Dataset	CXR	EXR	HCT	EEG
Comparable FS Size	Large	Large	Large	Medium
FS Labeling Time	9 Months	5 Months	3 Months	4 Months
DP Labeling Time	13 Hours	13 Hours	27 Hours	49 Hours
Percent of FS Time for DP	1.0%	1.8%	6.1%	7.9%

Table S3. Labeling times for comparable fully and weakly supervised models. We present labeling times for comparable models trained with full hand-labeled supervision (FS) and cross-modal data programming (DP). In each case, the DP model trained on the Large dataset is statistically equivalent to or superior to the FS model on the dataset size indicated by "Comparable FS Size." Labeling time for each case was computed by adding the time required to hand-label the development set to the time required to either hand-label (FS) or weakly label (DP) the training set. See Table 1 for dataset size definitions. Note that labeling times reported for full supervision are conservative, as we assume that only a single clinician contributed to reading each case.

		Text (Dev)			Image (Test)
Task	Model	F1 @ 0.5	ROC-AUC	Coverage	Median ROC-AUC
**CXR** (20 LFs)	FS	-	-	-	0.95 ± 0.005 (Med. 0.94)
	DM-GM	0.92 ± 0.006	0.95 ± 0.010	1.00	0.94 ± 0.005 (Med. 0.94)
	DM-MV	0.88 ± 0.064	0.96 ± 0.009	1.00	0.93 ± 0.007 (Med. 0.93)
	GM	0.86 ± 0.003	0.80 ± 0.005	0.88	0.88 ± 0.031 (Med. 0.89)
	MV	0.86	0.67	0.84	0.89 ± 0.019 (Med. 0.90)
**EXR** (18 LFs)	FS	-	-	-	0.91 ± 0.006 (Med. 0.90)
	DM-GM	0.83 ± 0.007	0.87 ± 0.006	1.00	0.92 ± 0.016 (Med. 0.94)
	DM-MV	0.80 ± 0.009	0.85 ± 0.011	1.00	0.87 ± 0.026 (Med. 0.88)
	GM	0.78 ± 0.001	0.84 ± 0.002	0.89	0.90 ± 0.018 (Med. 0.90)
	MV	0.82	0.80	0.77	0.88 ± 0.011 (Med. 0.88)
**HCT** (7 LFs)	FS	-	-	-	0.92 ± 0.027 (Med. 0.93)
	DM-GM	0.95 ± 0.006	1.00 ± 0.001	1.00	0.92 ± 0.037 (Med. 0.92)
	DM-MV	0.95 ± 0.004	1.00 ± 0.000	1.00	0.94 ± 0.023 (Med. 0.95)
	GM	0.96 ± 0.000	0.98 ± 0.000	1.00	0.90 ± 0.017 (Med. 0.90)
	MV	0.96	0.98	1.00	0.90 ± 0.048 (Med. 0.88)
**EEG** (11 LFs)	FS	-	-	-	0.92 ± 0.007 (Med. 0.92)
	DM-GM	0.87 ± 0.052	0.97 ± 0.010	1.00	0.84 ± 0.019 (Med. 0.85)
	DM-MV	0.81 ± 0.027	0.96 ± 0.008	1.00	0.83 ± 0.020 (Med. 0.84)
	GM	0.90 ± 0.000	0.96 ± 0.006	0.96	0.84 ± 0.023 (Med. 0.85)
	MV	0.88	0.95	0.95	0.84 ± 0.003 (Med. 0.84)

Table S4. Mean and variance of performance of different parts of the cross-modal data programming pipeline. We analyze the performance of majority vote of the labeling functions (MV), a generative model trained on these labeling functions (GM), a discriminative LSTM trained to map the raw text to the majority vote output (DM-MV), a discriminative LSTM trained to map the raw text to the generative model output (DM-GM), and hand-labeled full supervision (FS). We present F1 score at the default cutoff value of 0.5 to indicate how each model would perform as a binary classifier, and ROC-AUC to provide a more complete measurement of how well each model ranks positive and negative examples. Text model results are reported on the development (Dev) set, while downstream image model performance is reported on the held-out test (Test) set. ± represent standard deviations, and for image models we also present the median. Note that MV text model results and all coverage results are deterministic. We find that in cases where either generative model coverage or ROC-AUC is below 90%, the additional LSTM modeling step can improve text modeling performance substantially.

Supplemental Experimental Procedures

Details of Cross-Modal Data Programming Implementation for HCT

We provide a detailed description of how cross-modal data programming is implemented for the HCT application below.

We first curate and preprocess the dataset used for each application. For HCT, we create a dataset describing the binary task of intracranial hemorrhage detection by collecting 5,582 non-contrast HCT studies from our institution's Picture Archiving and Communications System (PACS) and procuring their associated text reports. We consider this problem within the *multiple instance learning* (MIL) framework, wherein an example should be considered abnormal if *any* of the individual frames contains evidence of hemorrhage.[43] We restrict our dataset to studies containing between 29 and 45 axial slices reconstructed at 5 mm axial resolution and retain the center 32 slices of each reconstruction, padding with images containing values of 0 Hounsfield Units where necessary. Clinicians then hand-label a small *development set*, which is used not as training data, but rather as an aid for tuning both clinician-provided LFs and model hyperparameters; for HCT, this required clinicians to label 170 HCT studies as positive or negative for hemorrhage.

As a second step, clinicians write LFs that for each report either provide a label or else abstain. For HCT, a single radiology fellow composed seven Python LFs over the text report based on their own experience reading and writing radiology reports, with several hours of support from a computer science graduate student. Third, we train a generative model to simultaneously learn the accuracies of all LFs. Concretely, we compute the Λ matrix by executing LF code to calculate output values for $m = 7$ LFs on $n = 4{,}000$ training examples, and then execute a single Python command in Snorkel[30] to estimate generative model parameters $\hat{\theta}$ by solving Eq. 1. Fourth, we assign a composite probabilistic label that represents an appropriately weighted combination of the LF outputs; practically, this translates into executing the trained generative model over each report. Clinicians then compare the output of the generative model to their ground truth development set labels, and repeat steps 2 - 4 until diminishing returns are observed with respect to generative model performance as evaluated against the development set. This entire procedure generally requires fewer than eight cumulative hours of clinician time per task.

We can next use our heuristic optimizer to determine whether or not to train an LSTM over the raw text to provide an augmented set of probabilistic labels. The inputs to this model would be the raw text reports, while the output targets would be the labels produced by the generative model. For HCT, the generative model labels have ROC-AUC and coverage of over 90% on the development set, so we bypass LSTM training and use the generative model labels directly as our source of weak supervision for our image model. Bypassing the LSTM training process in cases like this where we expect minimal performance improvement can save substantial amounts of computation time.

Once weak labels have been provided for a given task, a discriminative machine learning model (e.g. neural network classifier) can be trained over the raw data modality to evaluate the quantity of interest. For HCT, we define a standard attention mechanism over an 18-layer Residual network[9] encoder in PyTorch[11] that operates on every axial slice of the reconstructed tomographic image. This attention mechanism is a small neural network that dynamically learns how heavily a given slice should be weighted in the representation used by the final classification layer.[43] We then estimate optimal parameters $\hat{w}$ by approximating the solution to Eq. 2 using standard PyTorch backpropagation algorithms and a binary cross entropy loss function between the network prediction and the weak probabilistic label.

Computer Code for Cross-Modal Data Programming

We provide labeling function (LF) code for each medical imaging application and a functional demonstration of the entire cross-modal weak supervision technique on a small, public dataset at https://github.com/HazyResearch/cross-modal-ws-demo. The code provided in this demonstration exactly mirrors that used for the analysis presented in the manuscript.

Supplemental References

50. Asif, U., Roy, S., Tang, J., and Harrer, S. (2019). SeizureNet: Multi-Spectral Deep Feature Learning for Seizure Type Classification. arXiv Prepr. arXiv1903.03232.
51. Jnawali, K., Arbabshirani, M.R., Rao, N., and Patel, A.A. (2018). Deep 3D convolution neural network for CT brain hemorrhage classification. Proc. SPIE Med. Imaging 2018 Comput. Diagnosis *10575*, 105751C.
52. Roy, Y., Banville, H., Albuquerque, I., Gramfort, A., Falk, T.H., and Faubert, J. (2019). Deep learning-based electroencephalography analysis: A systematic review. J. Neural Eng. *16*.
